# All Three Subunits of RecBCD Enzyme Are Essential for DNA Repair and Low-Temperature Growth in the Antarctic *Pseudomonas syringae* Lz4W

**DOI:** 10.1371/journal.pone.0009412

**Published:** 2010-02-25

**Authors:** Theetha L. Pavankumar, Anurag K. Sinha, Malay K. Ray

**Affiliations:** Centre for Cellular and Molecular Biology, Council of Scientific and Industrial Research, Hyderabad, India; University of Hyderabad, India

## Abstract

**Background:**

The *recD* mutants of the Antarctic *Pseudomonas syringae* Lz4W are sensitive to DNA-damaging agents and fail to grow at 4°C. Generally, RecD associates with two other proteins (RecB and RecC) to produce RecBCD enzyme, which is involved in homologous recombination and DNA repair in many bacteria, including *Escherichia coli*. However, RecD is not essential for DNA repair, nor does its deletion cause any growth defects in *E. coli*. Hence, the assessment of the *P. syringae* RecBCD pathway was imperative.

**Methodology/Principal Findings:**

Mutational analysis and genetic complementation studies were used to establish that the individual null-mutations of all three genes, *recC, recB*, and *recD*, or the deletion of whole *recCBD* operon of *P. syringae*, lead to growth inhibition at low temperature, and sensitivity to UV and mitomycin C. Viability of the mutant cells dropped drastically at 4°C, and the mutants accumulated linear chromosomal DNA and shorter DNA fragments in higher amounts compared to 22°C. Additional genetic data using the mutant RecBCD enzymes that were inactivated either in the ATPase active site of RecB (RecB^K29Q^) or RecD (RecD^K229Q^), or in the nuclease center of RecB (RecB^D1118A^ and RecB^Δnuc^) suggested that, while the nuclease activity of RecB is not so critical in vivo, the ATP-dependent functions of both RecB and RecD are essential. Surprisingly, *E. coli recBCD* or *recBC* alone on plasmid could complement the defects of the *ΔrecCBD* strain of *P. syringae*.

**Conclusions/Significance:**

All three subunits of the RecBCD^Ps^ enzyme are essential for DNA repair and growth of *P. syringae* at low temperatures (4°C). The RecD requirement is only a function of the RecBCD complex in the bacterium. The RecBCD pathway protects the Antarctic bacterium from cold-induced DNA damages, and is critically dependent on the helicase activities of both RecB and RecD subunits, but not on the nuclease of RecBCD^Ps^ enzyme.

## Introduction

Bacteria living under extreme cold conditions of Antarctica have developed several adaptive features for growth and survival at low temperature [1]–[7]. We reported earlier that the inactivation of *recD* gene in the Antarctic psychrotrophic bacterium *Pseudomonas syringae* Lz4W leads to cold sensitivity [6]. The *recD* mutants of *P. syringae* are not only defective for growth at low temperature (4°C), but unlike in *Escherichia coli,* the mutants are also sensitive to DNA damaging agents (e.g., UV and mitomycin C). RecD polypeptide, encoded by *recD*, functions as a subunit of the hetero-trimeric RecBCD complex [8], also known as Exonuclease V (ExoV), in which RecD plays a regulatory role in activities of the complex. Enzymatically, RecBCD is a DNA-dependent ATPase with powerful helicase and processive exonuclease activities, and it has RecA loading activity onto 3′-ending single-stranded DNA (ssDNA) tail for homologous DNA pairing [9], [10]. The helicase/exonuclease activities of RecBCD have also been implicated in the degradation of foreign linear DNA. Among the two major DNA repair pathways (*recBCD* and *recFOR*) of bacterial cell, RecBCD machinery is primarily responsible for repairing the double stranded DNA breaks (DSBs). It helps in reestablishing the stalled or collapsed replication forks (RFs), by processing the broken double-stranded DNA (dsDNA) ends via linear DNA degradation and initiating the recombinational DNA repair that is largely regulated by a specific DNA sequence (5′-GCTGGTGG-3′) called χ (Chi, crossover hotspot instigator) on the *E. coli* chromosome [9]–[11].

Repair of chromosomal DSBs is crucial to cell survival during normal growth, as well as during assaults by exogenous DNA damaging agents. For this reason, the cultures of different *recB* and *recC* mutants including *recB* and *recC* null mutants of *E. coli* contain a large fraction of nonviable cells [12], and the mutants are sensitive to mitomycin C (MMC), UV and X-ray radiation [13], [14]. Temperature sensitive *recB* and *recC* mutants individually, or in combination, exhibit a temperature (42°C) induced drop in the cell viability, and additional *rep* inactivation in these thermo-sensitive mutants increase the accumulation of linear DNA fragments at high temperature [15], [16]. The RecBCD pathway is also known in protecting cells form nitric oxide induced DNA damage in *E. coli* [17] and H_2_O_2_ induced oxidative damage in *Neisseria gonorrhea* [18], and shown to be essential for the *Salmonella enterica* virulence in mice [19]. Taken together, it is becoming clear that faithful repair of damaged DNA by RecBCD dependent homologous recombination is essential for re-establishing the collapsed replication forks, as well as in the maintenance of genomic integrity under environmental conditions that cause DNA damage [20]. However, based on our studies with the *recD* mutants of Antarctic *P. syringae* it was not clear whether RecD requirement of the cold-adapted bacterium at low temperature is due to the functional inactivation of RecBCD complex or of the RecD protein alone [6]. Hence, the present genetic study was undertaken to investigate the importance of all three subunits in the RecBCD enzyme of *P. syringae* and analyze their roles in DNA damage repair and cold adaptation.

We report here that *recC*, *recB*, and *recCBD* deleted strains of *P. syrinage* are all severely growth defective at 4°C, but grow almost normally at 22°C. All these mutants are highly UV and MMC sensitive, and lose cellular viability at 4°C, similar to *recD* mutants. The mutants accumulate large amount of linear chromosomal DNA and shorter DNA fragments at 4° compared to 22°C. These defects in the mutants can be complemented by the respective wild-type genes of *P. syringae*, which were expressed from plasmid, suggesting that inactivation of any one of the subunits of RecBCD leads to functional inactivation of the whole protein complex. We also observed that the full complements, but not the individual subunits, of RecBCD from the psychrotrophic *P. syringae* (RecBCD^Ps^) and the mesophilic *E. coli* (RecBCD^Ec^) are exchangeable between the species for their requirement in cells. A little unexpectedly, we found that both the trimeric and dimeric enzymes of *E. coli*, RecBCD^Ec^ and RecBC^Ec^ (lacking RecD), were competent not only to protect the *ΔrecCBD* strain of *P. syringae* from UV and MMC treatment, but also in supporting the growth of the mutant at low temperature, suggesting that both RecBCD^Ec^ and RecBC^Ec^ complexes retain functional activity at 4°C. More importantly, the effects of specific active-site mutations (in ATP binding or nuclease catalytic sites) of RecB and RecD subunits suggests that the ATP-dependent helicase function is more crucial than the nuclease activity of RecBCD complex *in vivo*, and that the *recBCD* pathway of DNA repair is essential for low temperature adaptation of the psychrotrophic bacterium.

## Results

### Construction and Characterization of *recC*, *recB*, *recD* and *recCBD* Null-Mutants of *P. syringae*

**Figure 1:**
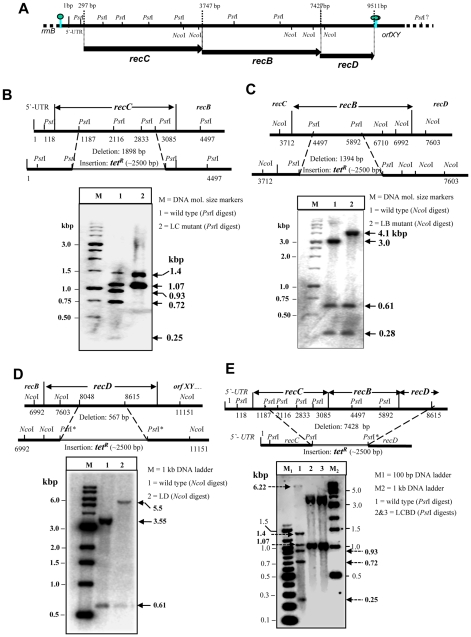
Disruption of genes in the *recCBD* operon and creation of LC *(ΔrecC*), LB (*ΔrecB*), LD (*ΔrecD*), and LCBD (*ΔrecCBD*) strains of *P. syringae* Lz4W. (**A**) Structural organization of the *P. syringae recCBD* region map (thick solid line) shown schematically with *Nco*I and *Pst*I restriction sites. The span of *recC*, *recB*, and *recD* reading frames have been indicated as dark arrowed boxes below the line. The numbers on map corresponds to the nucleotide position of the *recCBD* region sequence as reported (accession no AY078390). (**B**), (**C**), (**D**), and (**E**) depict the Southern hybridization results with LC, LB, LD, and LCBD genomic DNAs to confirm the gene deletions. On the top of B, C, D, and E panels, the extent of deletions and their replacement by the insertions of Tc-cassette (*tet^R^*) have been indicated schematically, with the locations of *Pst*I and *Nco*I restriction sites that were employed in the Southern analysis. (**B**) *Pst*I digested genomic DNAs of wild-type (wt) and LC were probed with ^32^P-labeled *recC* DNA as probe. Wild-type (lane 1) produced *recC* specific 1.4, 1.07, 0.93, 0.72 and 0.25 kbp DNA fragments. In LC (lane 2), about 1.9 kbp DNA of the *recC* comprising 0.93, 0.72 and 0.25 kbp fragments have been replaced by 2.5 kbp *tet^R^* gene, as expected for the double crossover mediated gene replacement. (**C**) *Nco*I digested wt and LB genomic DNAs were probed with ^32^P-labeled full-length *recB* gene. The 3 kbp DNA fragment in wt (lane 1) has given rise to 4.1 kbp DNA band in LB (lane 2) due to the replacement of 1.4 kbp of *recB* segment with 2.5 kbp Tc-cassette. (**D**) *NcoI* digested genomic DNAs of wt and LD strains were probed with ^32^P-labeled *recD* DNA. The 3.55 kbp DNA fragment in wt (lane 1) has given rise to ∼5.5 kbp DNA band in LD, due to the replacement of 567 bp *recD* DNA segment with 2.5 kbp *tet^R^*, as expected in a double cross over event. *Pst*I^*^ indicates the *Pst*I site that was created in the disruption plasmid vector, but not present on the wt chromosomal DNA. (**E**) *Pst*I digested genomic DNAs of wt and LCBD were probed with ^32^P-labeled *recCBD* DNA. In LCBD mutant (lanes 2 & 3, duplicated samples), a total of ∼7.42 kbp DNA segment of *recCBD*, which includes ∼1.4 kbp (two), 0.93, 0.72, and 0.25 kbp *Pst*I fragments in addition to 2.72 kbp DNA between the *Pst*I site at 5892 nt position of *recB* and *Pst*I^*^ site of *recD* at 8615 position were deleted. The 6.22 kbp *Pst*I DNA fragment in wild-type (lane 1) has reduced to ∼3.5 kbp in LCBD (lanes 2 & 3) due to the deletion of 2.72 kbp DNA. The 6.22 kbp DNA band shows lesser hybridization signal due inefficient transfer of the DNA onto the membrane blot.

The *recC*, *recB* and *recD* genes of *P. syringae* constitute a single operon of about ∼9.5 kbp DNA segment (Fig. 1A) [6]. The three overlapping reading frames for RecC, RecB, and RecD peptides are located on a common primary transcript. We generated LC (*ΔrecC*), LB (*ΔrecB*), LD (*ΔrecD*) strains of *P. syringae* (Table 1) by inactivating the respective genes individually, or deleting most of the *recCBD* operon in LCBD (*ΔrecCBD*), by replacement of the internal DNA segments in gene/s with a *tet^R^* gene (Tc-cassette) as described under Materials and Methods. A schematic of the gene replacements are depicted in the top panels of Figs. 1B through 1E. By Southern hybridization (Figs. 1B, C, D, E) and PCR analyses (data not shown) we confirmed the insertion of ∼2.5 kbp DNA of *tet^R^* into *recC*, *recB*, *recD*, and *recCBD* gene/s, and the deletion of 1898 bp from *recC*, 1394 bp from *recB,* 567 bp from *recD*, and 7428 bp from the *recCBD* operon in LC, LB, LD, and LCBD strains, respectively. Analysis also suggested that the chromosomal gene replacements have occurred through homologous recombination, by double crossover between the DNA segments provided on the suicidal plasmid-constructs and the chromosome of *P. syringae* (Fig. 1). We also confirmed the inactivation of the gene(s) of *recCBD* operon by Western analysis of the cellular proteins from LC, LB, LD and LCBD strains, using specific antibodies against the RecB, RecC, and RecD proteins (data not shown). As expected, LCBD strain lacked RecC, RecB, and RecD polypeptides, where as LB and LC strains were devoid of RecB and RecC, respectively.

**Table 1:** Bacterial and phage strains.

Strains	Genotype/characteristics	Source/reference
***Escherichia coli*** ** strain**		
V66	*argA21 hisG4 recF143 met rspL31 galK2 xyl-5 λ^−^ F^−^*	[25]
V67	As V66, plus *recB21::IS186*	[25]
V330	*Δ (recC- argA) 234 λ^−^ F^−^*	[25]
594	*lac-3350 galK2 galT22 rpsL179λ^−^ F^−^*	[25]
C600	*Thr-1 leuB6 thi-1 lacY1 tonA21 supE44 rfbD1 λ^−^ F^−^*	[25]
S17-1	*F^−^ pro recA1 (r^−^ m^−^) RP4-2 integrated (Tc::Mu) (Km::Tn7) [Smr Tpr]*; used as a plasmid mobilizing strain	[55]
***Pseudomonas syringae*** ** strain**		
*P. syringae* Lz4W	Wild-type, Antarctic isolate	[31]
LC	Δ*recC*:: *tet^r^*	This study
LB	Δ*recB*:: *tet^r^*	This study
LD	Δ*recD*:: *tet^r^*	This study
LCBD	Δ*recCBD*:: *tet^r^*	This study
**Phage strains**		
λ 872	*b1453 cI857*	[56]
λ 873	*b1453 χ^+^76 cI857*	[56]
λ 1081	*susJ6 b1453 cI857 χ^+^D123*	[35]
λ 1082	*b1453 χ^+^D123 susR5*	[35]
λ 1083	*susJ6 b1453 χ^+^76 cI857*	[35]
λ 1084	*b1453 χ^+^76 susR5*	[35]
T4	*gene 2^+^*	[22]
T4 2^−^	*gene 2 amN51*	[22]

### Growth Defects and Cell Death of LC, LB, LD, and LCBD Strains at Low Temperature

**Figure 2:**
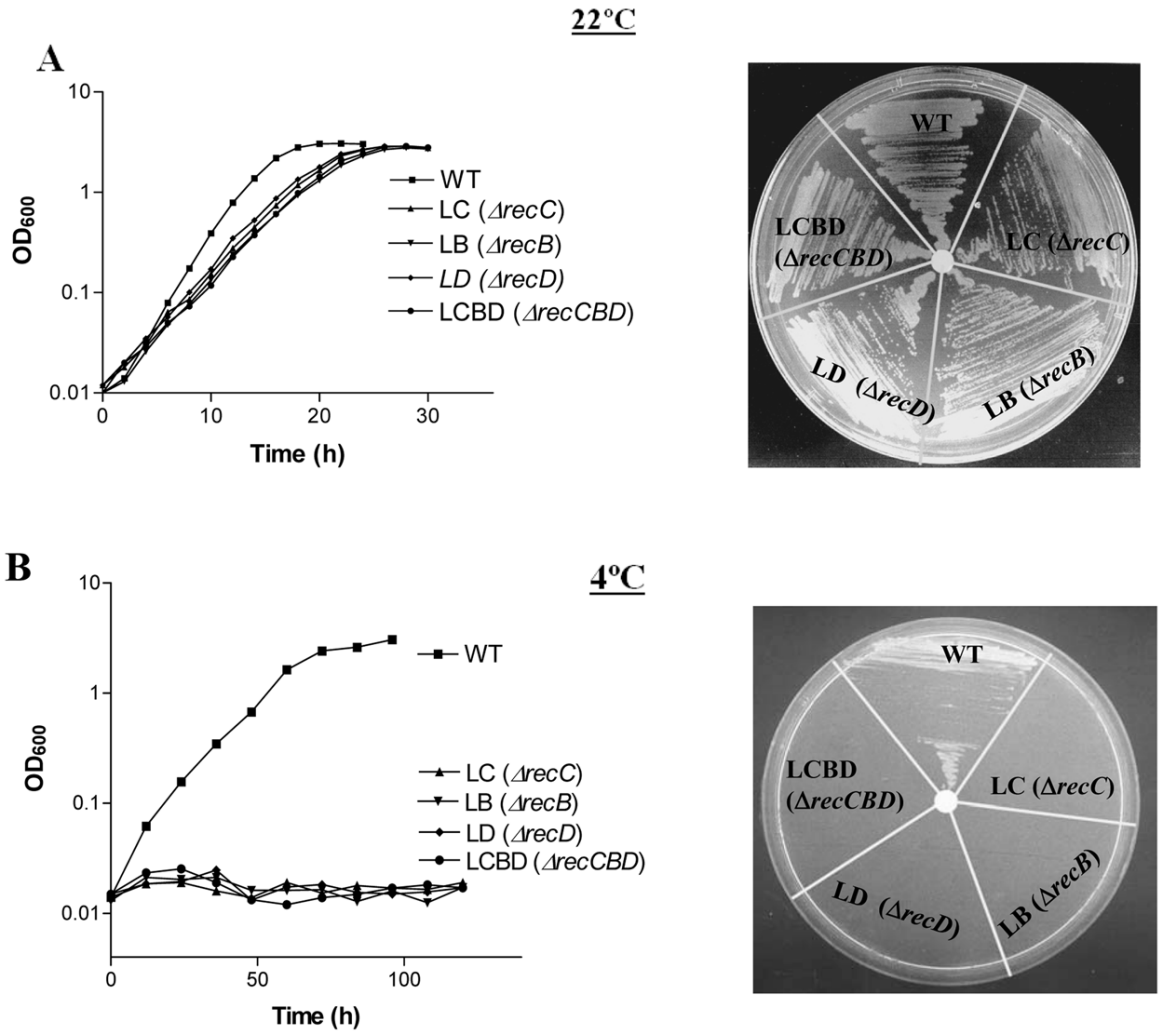
Growth analysis of wild-type and the *recCBD* mutants (LC, LB, LD, and LCBD) of *P. syringae*. The growth was assessed at 22°C (**A**) and at 4°C (**B**), both in ABM broth (left panels) and on ABM-agar plate (right panels).

Growth profiles of wild-type (WT) and the *recC*, *recB*, *recD* and *recCBD* null-mutants were checked by growing the strains in liquid broth at 22° and 4°C. The mutant strains LC, LB, LD and LCBD grew almost normally at 22°C, albeit at a slightly slower rate compared to the wild-type (Fig. 2A). Generation time (3.2 to 3.7 hr) of the mutants in general was relatively longer compared to that of wild-type (∼2.5 hr). However, at 4°C, all four mutants were severely growth defective, and completely failed to grow both in liquid broth and on ABM-agar plates (Fig. 2B).

**Figure 3:**
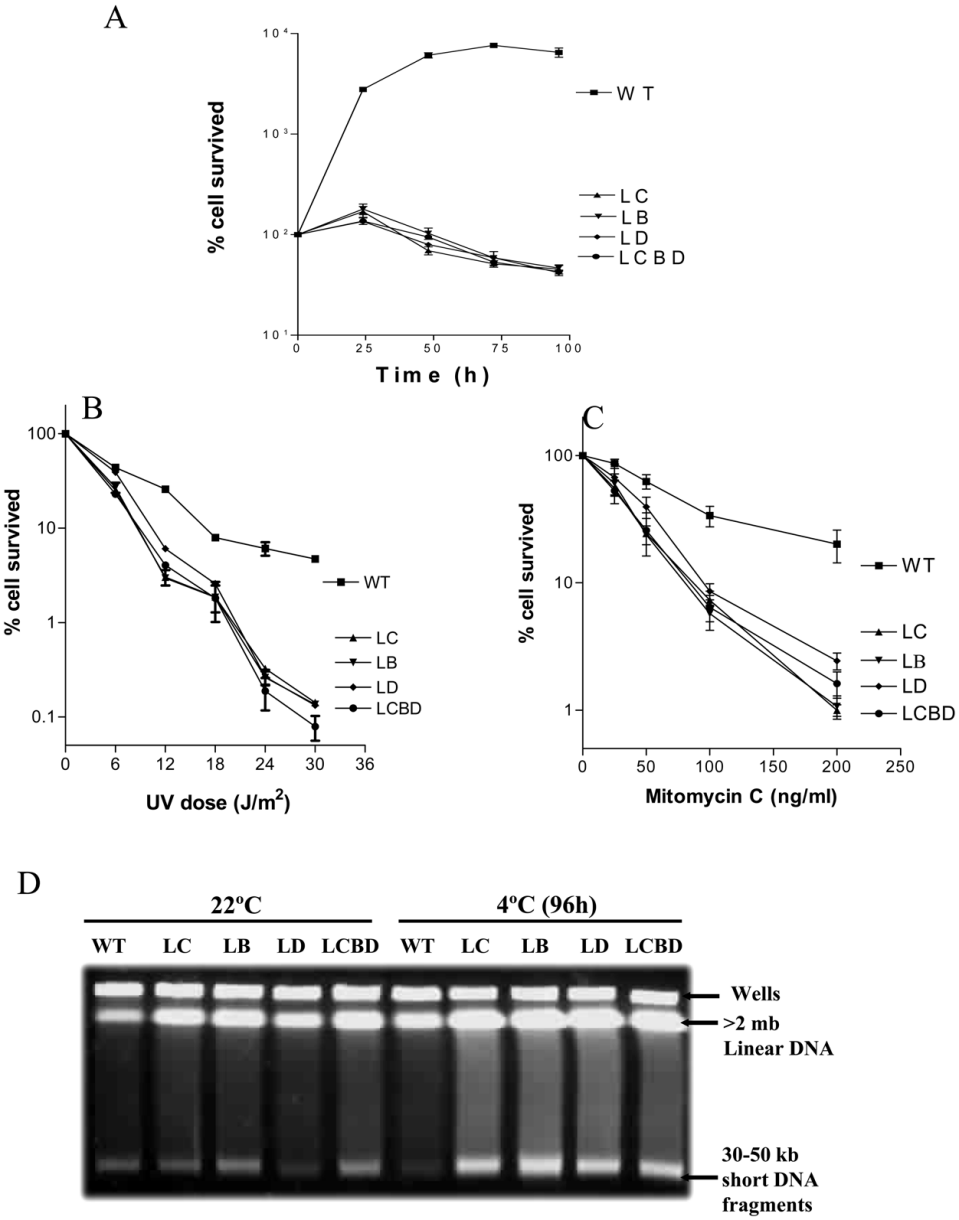
Cell survival and sensitivity to UV and mitomycin C (MMC) of wild-type (wt) and *recCBD* mutants (LC, LB, LD, and LCBD). (**A**) Cell viability was assessed by shifting the 22°C grown cultures to 4°C, and then measuring the colony forming ability of cells on ABM-agar plates at every 24 hrs. The number of cells (cfu) just before the shift (0 time point) in each culture was considered as 100%. Sensitivity to UV (**B**) and MMC (**C**) was assessed at 22°C following the method described under Materials and Methods. For UV irradiated experiments, cell survival was calculated by considering the cfu of unirradiated cells as 100%. For MMC sensitivity tests, cells were incubated with different concentration of mitomycin C for 30 min, washed, serially diluted and plated for colony development. The cell survival was calculated by considering the cfu of untreated cells as 100%. (**D**) PFGE analysis of DNA damage in wt and mutants. Cells (22°C grown, or 96 hr post-shift at 4°C) were processed for PFGE as described under Materials and Methods. Each lane contains ∼0.5×10^7^ cells. Wells containing circular chromosomal DNA, in-gel linear chromosomal DNA (labeled as >2 mb), and the short DNA fragments that make prominent band at ∼30–50 kbp region of the gel have been labeled.

Since *recBC* mutants of *E. coli* are generally ∼28% viable, due to the segregation of a high fraction of nonviable progeny, when grown under the laboratory conditions [12], we examined the situation in *P. syringae*. We compared the viability of LC, LB, LD and LCBD cells with that of wild-type, by enumerating the colony forming unit (cfu) of cells in the cultures grown at 22°C. We found that the cfu of all four mutant strains in the cultures were about 30% of the wild-type. We then examined the viability of mutant cells by shifting the 22°C grown cultures (∼0.5 OD_600_) to 4°C. After about 48 hours at the low temperature, the number of viable cells (cfu) in the cultures started dropping, and by about 96 hr the number was reduced to 40% of the cells that existed before the shift (Fig. 3A). Thus, the viability of the *recBCD* mutant cells at 4°C appears to be very poor; only about 12% cells (i.e., 40% of the 30% cells that survived in the 22°C grown cultures) compared to the wild-type retained the colony forming ability. Increased cell death of the mutant cells at 4°C was further confirmed by fluorescent labeling of the live and dead cells, as described under Materials and Methods. The proportion of Syto-9 stained live cells decreased, and the propidium iodide (PI) stained dead cells increased in the cultures at 4°C (Supporting Information S1). We also observed that size of the *recB* and *recC* mutant cells at 4°C was relatively larger, compared to WT cells (Supporting Information S1), as noted earlier for the *recD* mutants of *P. syringae* [6].

### LC, LB, LD, and LCBD Strains Are Sensitive to UV and Mitomycin C, and Also Accumulate Damaged DNA in Cells at Low Temperature

To assess the importance of the individual subunits of RecBCD protein complex in the recombinational repair of DNA, we tested the effects of DNA damaging agents, UV and MMC, on LC, LB, LD and LCBD strains of *P. syringae*. All the four mutants displayed high levels of sensitivity to the DNA damaging agents (Figs. 3B, 3C), compared to the wild-type, as reported earlier for the transposon induced *recD* mutant (CS1) of *P. syringae* [6]. There was not much difference in the degrees of sensitivity among the mutants with single gene deletions or the whole operon deletion, thus suggesting their role in a common pathway.

Fig. 3D shows the pulsed field gel electrophoresis (PFGE) analysis of cellular DNAs from the LC, LB, LD and LCBD strains, following the shift of bacterial cultures from 22° to 4°C. PFGE was performed to examine the status of chromosomal DNA in cells. Generally, linear chromosomal DNA under the PFGE conditions enters into the gel, whereas circular intact chromosomal DNA remains in the wells [16], [21]. We observed that all four mutants accumulated linear chromosomal DNA, and short DNA-fragments (prominent around 40–50 kb size region) in larger amount, compared to the wild type, at both 22°C and 4°C. However, the amount of linear chromosomal DNA and short DNA fragments was significantly higher at 4°C compared to 22°C (Fig. 3D), as reported earlier in the case of transposon induced *recD* mutant strain CS1 of *P. syringae* [6].

### Genetic Complementation of the Mutants with Wild-Type Genes

**Figure 4:**
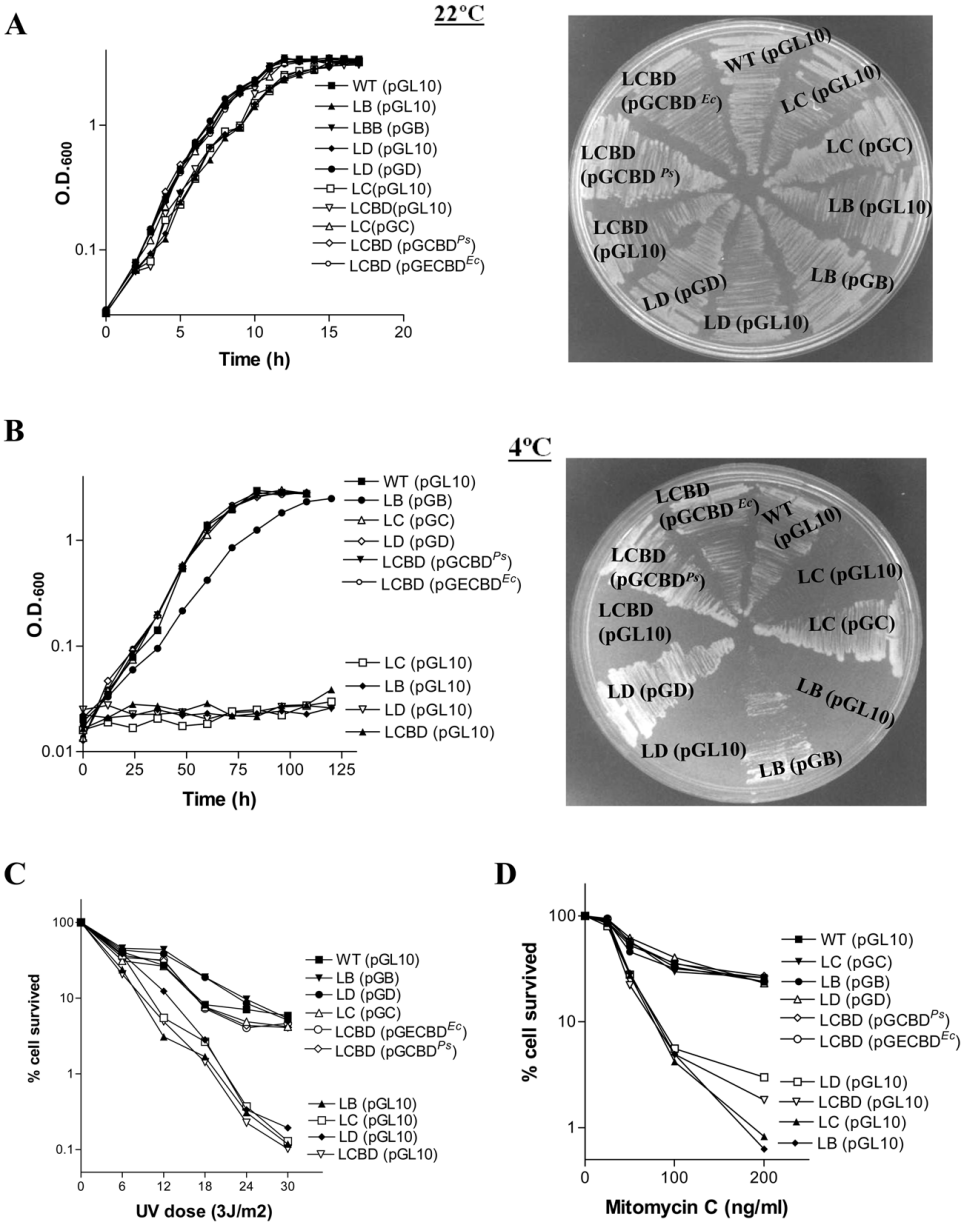
Genetic complementation of *recBCD* mutants of *P. syringae*. Growth analysis of the WT, mutant strains (LC, LB, LD, and LCBD), and the mutants bearing the plasmid-borne individual wild-type genes (*recC* on pGC, *recB* on pGB, *recD* on pGD) or all three genes (pGCBD^Ps^ for *P. syringae* and pGECBD for *E. coli* genes), or the empty plasmid vector (pGL10) were carried out at 22°C (**A**) and 4°C (**B**), both in ABM broth (shown in left) and on agar plate (shown in right). Plasmids carried by the strains have been indicated within the brackets in each case. The results of UV (**C**) and mitomycin C (**D**) sensitivity tests are shown for wild-type, different *recBCD* mutants, and the complemented mutants harboring the respective plasmid borne genes.

Although above results suggest that all three genes of the *recCBD* operon are important for DNA repair process in the bacterium, a polar effect on the expression of downstream genes could not be ruled out for the mutant phenotypes. Therefore, the ability of each individual genes of *recCBD* operon was tested by expressing them from plasmids in *trans* to rescue the growth defects of LC, LB, LD, and LCBD strains. The plasmid-borne genes encoded the wild-type 6xHis-tagged proteins of RecB, RecC, and RecD of *P. syringae* (Table 2). The analysis (Fig. 4) shows that the defects of the mutants are abolished in the presence of respective wild-type proteins. The complemented mutants not only gained the capacity to grow at 4°C (Figs. 4B), but also acquired resistance to UV and mitomycin C, similar to the wild-type (Figs. 4C, D). Furthermore, their cell-viability in the cultures was also restored to the wild-type level (Supporting Information S1). Additionally, the complemented mutant strains exhibited reduced accumulation of both linear chromosomal DNA and shorter DNA fragments in the cells, as evidenced by PFGE analyses (Supporting Information S1), confirming that inactivation of any single gene of the *recCBD* operon leads to the observed defects in cells. It is also clear that LC, LB, LD and LCBD strains are deficient only in *recC, recB, recD* and *recCBD* gene/s functions, respectively. The lack of polar effects, due to insertion of the *tet^R^*-cassette in *recC* and *recB* genes on the expression of downstream genes of *recCBD* operon in LC and LB mutants, suggests that there might be additional internal promoters lying within the residual 3′end DNA segments of *recC* (662 bp) and of *recB* (1.535 kbp) genes respectively, which drive the expression of downstream *recB* and *recD* genes in the mutants.

**Table 2:** Plasmids and their derivatives used in this study.

Plasmid	Description / characteristics	Reference
pGL10	Broad-host cloning vector, IncP replicon, *mob^+^, Km^r^*	[57]
pGC	*P. syringae recC* gene cloned in pGL10 (produces N-terminal His-tagged RecC)	This study
pGB	*P. syringae recB* gene cloned in pGL10 (produces N-terminal His-tagged RecB)	This study
pGD	*P. syringae recD* gene cloned in pGL10 (produces C-terminal His-tagged RecD)	[52]
pGCBD	9.5 kbp of *P. syringae recCBD* operon in pGL10 (produces His-tagged RecC, and RecB and RecD proteins)	This study
pGCB^K28Q^ D	As pGBCD, but contains *recB^K28Q^* allele of *recB* gene	This study
pGCB^D1118A^D	As pGBCD, but contains *recB^D1118A^* allele of *recB* gene	This study
pGCBD^K229Q^	As pGBCD, but contains *recD^K229Q^* allele of *recD* gene	This study
pGB^Δnuc^	Deleted pGB construct producing truncated RecB peptide (1-1062 amino acid) lacking C-terminal 165 residues (1063–1227 amino acid) of nuclease domain	This study
pMMB206	Broad-host cloning vector, IncQ replicon, *Cm^r^*	[58]
pMJ	*P. syringae recJ* gene cloned in pMMB206	This study
pFS-11-04	18.5 kbp *Bam*HI fragment of *E. coli* chromosome containing *recCBD* genes between *thyA – argA* region in pBR322	[53]
pGECBD	18.5 kb *Bam*HI fragment of pFS-11-04 containing *E. coli recCBD* genes cloned in pGL10	This study
pAMP3	11.7 kbp DNA of *E. coli recC-ptr-recB* region in pSC101	[54]
pGECB	11.7 kbp DNA of *E. coli recC-ptr-recB* from pAMP3 cloned in pGL10	This study
pJQ200SK	Suicidal plasmid vector for *Pseudomonas* species, *mob^+^ Gm^r^*	[50]
pMOS^tet^	∼2.5 kb *Pst*I fragment bearing *Tet^r^* gene cassette in pMOS*Blue*	This study
pJQC^tet^	890 bp 5′end - Tc-cassette - 663 bp 3′end of *recC* gene in pJQ200SK, *Gm^r^, tet^r^*; a suicidal construct for *recC* disruption	This study
pJQB^tet^	754 bp 5′end - Tc-cassette - 1535 bp 3′end of *recB* gene in pJQ200SK, Gm^r^, tet^r^; a suicidal construct for *recB* disruption	This study
pJQD^tet^	670 bp 5′end - Tc-cassette - 1000 bp 3′end of *recD* gene in pJQ200SK, Gm^r^, tet^r^; a suicidal construct for *recD* disruption	This study
pJQCBD^tet^	890 bp 5′end of *recC* - Tc-cassette-1000 bp 3′end of *recD* in pJQ200SK, Gm^r^, tet^r^; suicidal plasmid for *recCBD* disruption	This study

**Figure 5:**
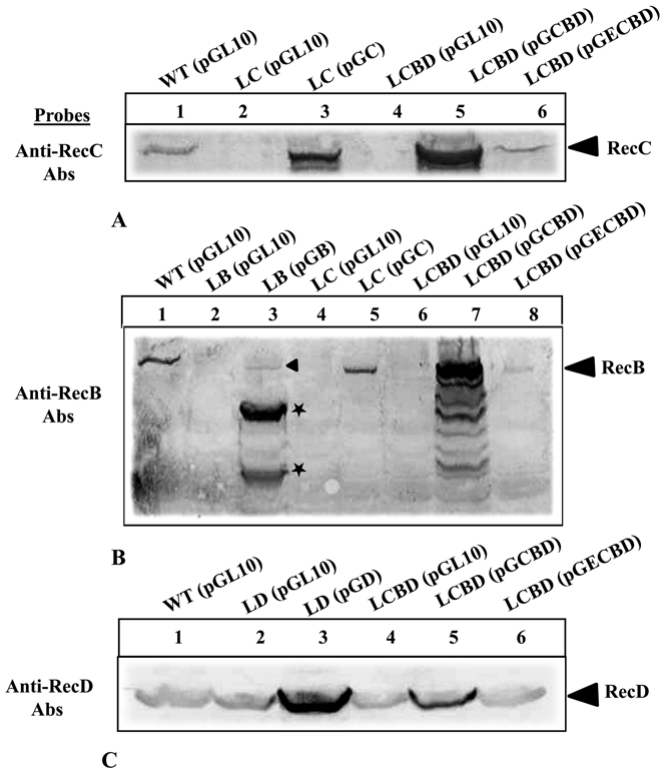
Western analysis of RecBCD expression in the wild-type and complemented mutants. Proteins in the cell extracts of 4°C grown strains were separated on SDS-PAGE and probed with specific polyclonal antibodies (Abs) raised against RecC, RecB and RecD polypeptides. (**A**) *P. syringae* RecC peptide specific bands is visible only in the producing strains (lanes 1, 3, and 5) but not in the *recC* deleted strains (lanes 2 and 4). *E. coli* RecC protein, expressed from multicopy plasmid pGECBD, cross-reacted to the anti-RecC Abs only weekly (lane 6). (**B**) Anti-RecB Abs cross-reacted to *P. syringae* RecB peptide in WT, LB harboring pGB, LC, and LCBD complemented strain (lanes 1, 3, 5, and 7 respectively). Over-expressed RecB from plasmid degraded very fast (lane 3) and hence shows lesser amount of intact peptide (marked by arrow head) and high amount of two degraded products (marked by *). *E. coli* RecB reacts very poorly to the anti-RecB Abs (lane 8). (**C**) Anti-RecD Abs cross-react to RecD peptides, which are easily distinguishable over the background when RecD is expressed from multicopy plasmid (lanes 3 and 5) but not in WT (lane 1) due to an unknown protein which cross-react to the RecD antisera (lanes 2, 4, and 6).

Western analyses were performed to confirm the expression of the proteins in the complemented strains. The analysis indicated that the levels of RecB, RecC and RecD peptides in these strains were higher than in the wild-type (Fig. 5). We also noticed that the RecB when expressed in excess degrades very fast (lanes 3 and 7, Fig. 5B), and that RecB is produced in LC cells (lane 5, Fig. 5B) in spite of the disruption of upstream *recC* in the *recCBD* operon. The analysis of RecD expression in cells by Western analysis using anti-RecD antibodies was not foolproof, due to immuno-crossreactivity of an unknown protein at the same region of polyacrylamide gel, where the RecD peptide migrates (Fig. 5C). However, RecD peptide when present in higher amount (e.g., produced from plasmid) generate prominent band over the background upon cross-reaction to the RecD antibodies (lanes 3 and 5, Fig. 5C). Interestingly, the *E. coli* RecBCD proteins which were also tested for complementation did not cross-react or reacted very poorly to the antibodies raised against the *P. syringae* protein subunits.

### Recombination Proficiency and Exonuclease Activity of the *P. syringae* RecBCD Enzyme and Its Mutants

The lack of RecBCD dependent DNA repair is most likely responsible for the cell death and the low-temperature growth defects of LC, LB, LD and LCBD mutants. During the repair of dsDNA breaks, broken DNA ends are processed by the ATP-dependent combined helicase and nuclease activities of RecBCD enzyme leading to the generation of 3′-extended tail of ssDNA, onto which the RecBCD loads RecA protein to produce ssDNA-RecA filament enabling it to pair with intact homologous DNA for recombination. Therefore, it was important to determine which of the multiple enzymatic activities of RecBCD is responsible for the DNA repair defect at low-temperature. This approach requires the generation of mutations in *recCBD* genes that lead to inactivation of some of these enzymatic activities in RecBCD complex, in addition to the availability of a genetic system for assessing the activities. Owing to the lack of a suitable system in *P. syringae* Lz4W, we determined the activities of RecBCD^Ps^ in the surrogate system of *E. coli* that lacked the endogenous RecBCD activity (e.g., V67 and V330 strains in Tables 3 and 4). In *E. coli*, the RecBCD activities are conveniently determined using phage multiplication assays for exonuclease V (ExoV), and the marker exchanges on chromosome or on λ phage DNA for recombination proficiency [22].

**Table 3:** Recombination proficiency and Chi activity of the *P. syringae* RecBCD enzymes.

*E. coli* Strain	*rec* alleles^a^	Hfr recombination (% His^+^ [Str^R^])^b^	λ phage recombination (% J^+^R^+^ recombinants)^c^		Chi activity^d^
			Cross 1	Cross 2	
V66(pGL10)	*recBCD^Ec^*	3.7±0.1	*9*±*0*	9.55±0.15	5.3±1.15
V67(pGL10)	none	0.003±0.00035	0.135±0.025	0.165±0.015	1.045±0.075
V67(pGECBD)	*recBCD^Ec^*	3.3±0.1	7.28 ±0.92	9.325±2.025	5.48±0.785
V67(pGCBD)	*recBCD^Ps^*	1.7±0.3	5.45±0.25	5.1±0.70	0.955±0.155
V67(pGECB)	*recBC^Ec^*	3.28±0.785	8.7±1.3	7.25±0.25	1.01±0.13
V67(pGCB)	*recBC^Ps^*	0.002±0.0004	0.44±0.06	0.535±0.045	1.035±0.085
V67(pGCB^K28Q^D)	*recB^K28Q^CD*	0.002±0.00015	0.330±0.015	0.465±0.005	1.16±0.14
V67(pGCB^D1118A^D)	*recB^D1118A^CD*	0.003±0.001	0.305±0.015	0.33±0.04	1.29±0.25
V67(pGCBD^K229Q^)	*recBCD^K229Q^*	0.002±0.001	0.455±0.105	0.405±0.235	1.23±0.23

a The enzyme alleles were expressed from plasmid-borne genes, except in V66 strain that had wild-type *recBCD* alleles on chromosome. The mutant enzyme alleles (*recB^K28Q^CD*, *recB^D1118A^CD*, and *recBCD^K229Q^*) were all derivatives of the *P. syringae* enzyme (*recBCD^Ps^*).

b The recombination frequency values are based on three independent crossings between the recipient V67 containing the plasmid-borne *recBCD* alleles and donor strain V1306 (Hfr PO44). The standard error of means (± SEM) have been indicated.

c The frequency of J^+^R^+^ recombinants in each set of crosses (Cross 1: phage 1081×1082, and Cross 2: phage 1083×1084) was determined as described under Materials and Methods.

d Data are the mean ± SEM from three independent experiments.

**Table 4:** Exonulclease V activity of the *P. syringae* RecBCD enzymes in T4 phage assays.

*E. coli* Strain	*rec* alleles^a^	T4 2^−^ phage titer^b^	T4 phage titer^c^
V66(pGL10)	*recBCD^Ec^*	0.96×10^−6^	1.22
V330(pGL10)	none	1.0	1.0
V330(pGECBD)	*recBCD^Ec^*	0.95×10^−6^	0.90
V330(pGCBD)	*recBCD^Ps^*	0.65×10^−6^	1.03
V330(pGCB^K28Q^D)	*recB^K28Q^CD*	0.83×10^−3^	0.77
V330(pGCB^D1118A^D)	*recB^D1118A^CD*	1.05	1.38
V330(pGCBD^K229Q^)	*recBCD^K229Q^*	0.51×10^−3^	1.2

a *recBCD* alleles were present on the plasmids, except in V66 strain that had the wild type *recBCD* alleles on chromosome.

b T4 2^−^ phage titer on the indicated strain divided by the titer on V330 (E.O.P. ∼3×10^10^/ml).

c T4 phage titer on the indicated strain divided by the titer on V330 (E.O.P. ∼2.7×10^11^/ml).

The values are average of two independent experiments.

**Figure 6:**
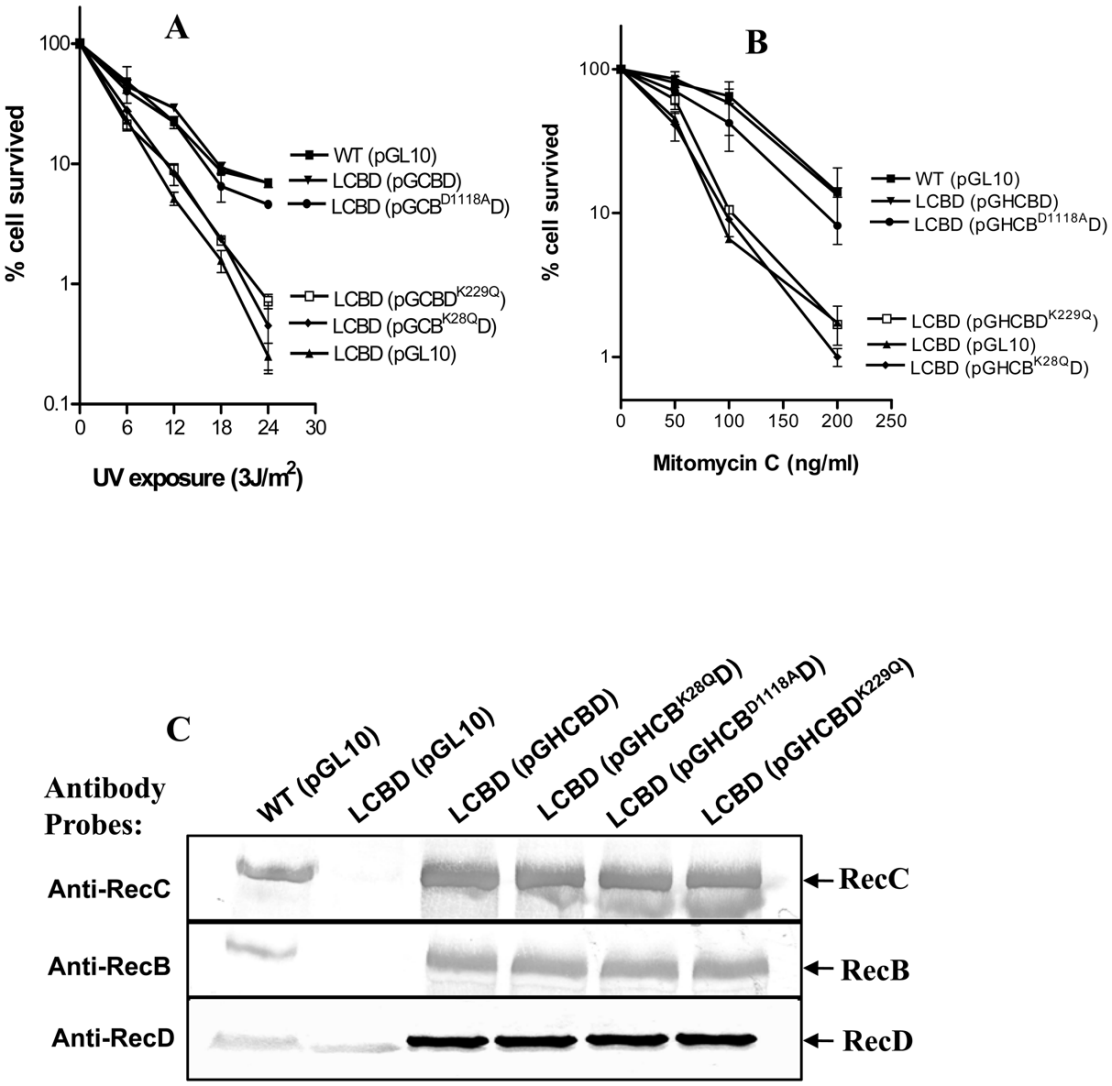
DNA repairing proficiency of RecB^K28Q^CD, RecB^D1118A^, and RecBCD^K229Q^ enzymes of *P. syringae*. LCBD (*ΔrecCBD*) strains carrying the plasmid-borne mutant alleles of *recB* (*recB^K28Q^* or *recB^D1118A^*) or *recD* (*recD^229Q^*) of *recCBD* operon were tested for UV (**A**) and mitomycin C (**B**) sensitivity, and compared with the wild-type (WT) harboring the empty plasmid vector (pGL10). (**C**) Western analysis of RecBCD protein production in WT and LCBD strains harboring the indicated plasmids. Polyclonal antibodies (raised against the RecC, RecB and RecD polypeptides) which were used as probes in the analysis have been indicated at left of the three panels.

We expressed the heterotrimeric RecBCD enzymes of *P. syringae* (RecBCD^Ps^) and *E. coli* (RecBCD^Ec^) as well as the dimeric complexes (RecBC^Ps^ and RecBC^Ec^) in V330 or V67 strains of *E. coli*. In addition, we separately created single amino acid substitution of the conserved residues in the ATP binding sites of both RecB (RecB^K28Q^) and RecD (RecD^K229Q^) subunits (Supporting Information S1). These point mutations inactivate the DNA-stimulated ATP hydrolysis and hence helicase activity of the respective subunits, leading to the formation of defective RecB^K28Q^CD and RecBCD^K229Q^ enzymes in cells. Another mutation was created in the conserved aspartic acid residue of *P. syringae* RecB at 1118 position (D1118) corresponding to the D1080 residue of the nuclease catalytic center of *E. coli* RecB (Supporting Information S1), which resulted in the change of aspartic acid to alanine (RecB^D1118A^). In *E. coli* the corresponding substitution (D1080A) produces a defective RecB^D1080A^CD enzyme that lacks nuclease and RecA-loading activity affecting the recombination and DNA repair proficiency of cells [23], [24]. We first confirmed the mutations in *recB* and *recD* by DNA sequence analysis of the respective mutant gene constructs, and the expression of mutant enzymes (RecBCD^Ps^, RecB^K28Q^CD, RecBCD^K229Q^, RecB^D1118A^CD, and RecBC^Ps^) by Western analysis, using antisera against the subunits (Fig. 6).

Recombination proficiency of the wild-type and mutant RecBCD enzymes were then assessed by the Hfr conjugational crosses, and in mixed phage lambda (λ) infection assays in V67 (*recB21::IS186*) strain of *E. coli* as shown in Table 3. RecBCD^Ps^ was found to be functionally active in *E. coli*. However, the frequency of His^+^ recombinants was about half (1.7%) in the presence of RecBCD^Ps^, compared to the wild-type RecBCD^Ec^ enzyme. In the λ phage recombination assay too, the RecBCD^Ps^ enzyme exhibited lesser efficiency than the *E. coli* enzyme (Table 3). RecBCD^Ps^ also failed to recognize the *E. coli* Chi (χ), as there was no increase in the Chi-stimulated recombination frequency as seen with the RecBCD^Ec^ enzyme in the λ phage recombination assays. It is also clear from Table 3 that the recombination frequency observed in *E. coli* cells expressing RecBC^Ps^ (i.e., the enzyme lacking RecD subunit) was almost negligible (0.002%) in contrast to the RecBC^Ec^(-D) enzyme, which is highly proficient in recombination [25]. The recombinational inactivity of RecBC^Ps^ enzyme is consistent with our observations that *recD* inactivated strains LD and CS1 [6] are sensitive to UV or MMC due to the lack of recombinational DNA repair (Fig. 4). More importantly, we now demonstrate that an inactive RecD^K229Q^ subunit (i.e., RecBCD^K229Q^ enzyme) or an inactive RecB subunit (i.e., RecB^K28Q^CD) also makes the *P. syringae* RecBCD enzyme inefficient in homologous recombination (Table 3). In addition, we observed that RecB^D1118A^CD enzyme with defect in the nuclease center of RecB lacks recombination proficiency in the surrogate *E. coli* system. This was unexpected as the *recB^D1118A^* allele was efficient in the DNA repair assay in *P. syringae* (see below).

The λ phage plaque assays on the lawn of V330 cells of *E. coli* also produced some unexpected results. While λ Red^-^ Gam^-^ χ^+^ phages made large plaques on the lawn of V330 expressing RecBCD^Ps^ and RecBCD^Ec^ enzymes, the λ Red^-^ Gam^-^ χ° phages produced small plaques only in the presence of RecBCD^Ec^ but not in the presence of RecBCD^Ps^. The small plaque-size occurs due to the ability of wild-type RecBCD enzyme to block the λ rolling-circle replication activation [22], which was obviously found lacking in the otherwise active RecBCD^Ps^ enzyme. In the presence of inactive *P. syringae* enzymes (RecB^K28Q^CD, RecB^D1118A^CD, and RecBCD^K229Q^) too, λ Red^-^ Gam^-^ χ° phages produced large plaques, suggesting that the λ plaque size assay cannot differentiate between the inactive and active *P. syringae* enzymes.

The nuclease (ExoV) activity of RecBCD enzymes was determined by the T4 2^-^ phage multiplication assay [26]. As evident from Table 4, RecBCD^Ps^ is as efficient an exonuclease as is the *E. coli* enzyme (RecBCD^Ec^). In contrast, the RecB-nuclease mutant enzyme (RecB^D1118A^CD) of *P. syringae*, as expected, was highly deficient in the ExoV activity. Interestingly, the mutant enzymes (RecB^K28Q^CD and RecBCD^K229Q^) with alteration in the ATPase active sites of RecB and RecD also displayed about 1,000-fold reduced activity of ExoV in the T4 2^-^ phage-multiplication assay. The residual ExoV activity of RecB^K28Q^CD and RecBCD^K229Q^ were however 1000-fold more efficient than the RecB^D1118A^CD in the *in vivo* exonuclease assays.

### DNA Repairing Competence and Growth Supporting Ability of RecB^K28Q^CD, RecB^D1118A^CD, and RecBCD^K229Q^ Enzymes

To test the importance of nuclease and helicase activities in the DNA repair and in supporting the growth at low temperature, the RecB^K28Q^CD, RecB^D1118A^CD, and RecBCD^K229Q^ enzymes were tested following their expression from plasmids in the *ΔrecCBD* strain of *P. syringae*. As expected, LCBD cells expressing RecB^K28Q^CD, RecBCD^K229Q^ enzymes remained sensitive to UV and mitomycin C. The cells containing RecB^D1118A^CD enzyme, on the other hand, conferred resistance to the DNA damaging agents, almost comparable to wild-type enzymes (RecBCD^Ec^ and RecBCD^Ps^) (Fig. 6A, B). This suggested that LCBD cells do not depend on the nuclease activity of RecB^D1118A^CD enzyme for the repair of UV and MMC induced DNA damages *in vivo*.

**Figure 7:**
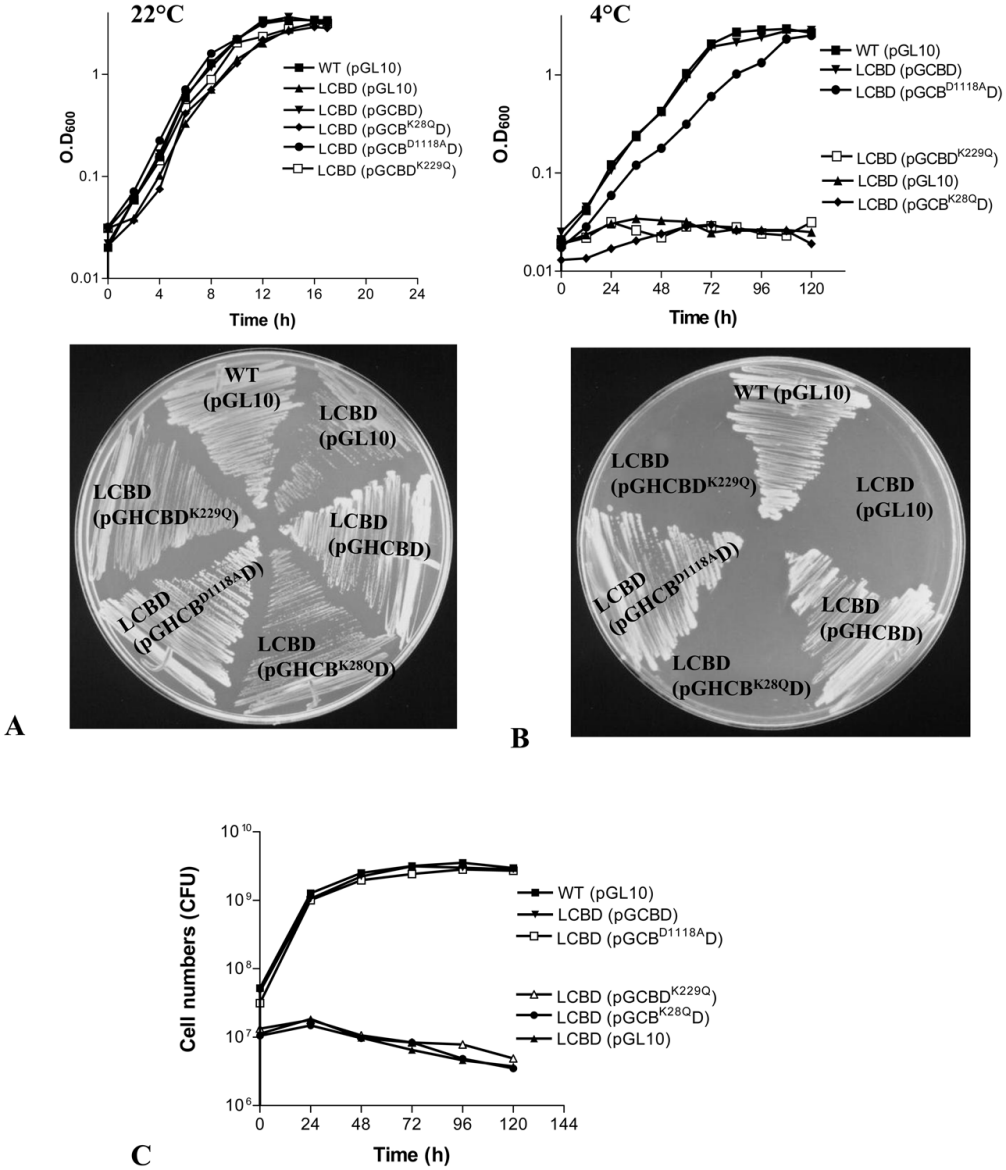
Growth and cell viability of LCBD expressing RecB^K28Q^CD, RecB^D1118A^, and RecBCD^K229Q^ enzymes. The RecBCD enzymes were produced in LCBD strain from the plasmid borne *recCBD* alleles as described in Fig. . The growth of the strains analyzed in ABM broth or on ABM-agar plates have been shown for 22°C (**A**) and 4°C (**B**). Cell survival (**C**), following the shift from 22°C to 4°C, were assessed by determining the number of cells (cfu) in the cultures at different time points as described under Materials and Methods.

The mutant enzymes were also tested for their ability to restore the growth defects of LCBD strain at 4°C (Fig. 7). The LCBD (pGHCB^D1118A^D) regained the ability to grow at the low temperature, similar to the wild-type strain. The inactivated alleles of RecB (*recB^K28Q^*) and RecD (*recD^K229Q^*) from similar constructs (*viz.*, pGHCB^K28Q^D and pGHCBD^K229Q^), however, failed to complement the cold-sensitivity of LCBD. Thus, it appears that recombination-deficient mutant enzymes, such as RecB^K28Q^CD and RecBCD^K229Q^, which are inefficient in the DNA repair, do not also support the growth of *P. syringae* at 4°C. However, the apparently recombination-deficient RecB^D1118A^CD enzyme (Table 3) was found to be proficient in DNA repair (Fig. 6) and able to restore the growth of LCBD at low temperature (Fig. 7). This lack of correlation between the recombination proficiency and the DNA repair exhibited by the RecB^D1118A^CD enzyme is likely due to the functioning of a hybrid pathway comprising of *recBCD* and *recF*-dependent repair systems of *P. syringae*, as has been observed in *E. coli* [27]–[30]. The *E. coli* V67 and V330 strains that were employed in the present study for the recombination activity assay (Table 3) are deficient in the *recF* pathway, as pointed out earlier [29]. The recombination incompetency of RecB^D1118A^CD enzyme of *P. syringae* in the surrogate *E. coli* strains possibly is likely due to the host *recF* inactivation.

### *recJ* on a Multicopy Plasmid Rescues the Slower Growth of *recB^Δnuc^* Strain at Low Temperature

**Figure 8:**
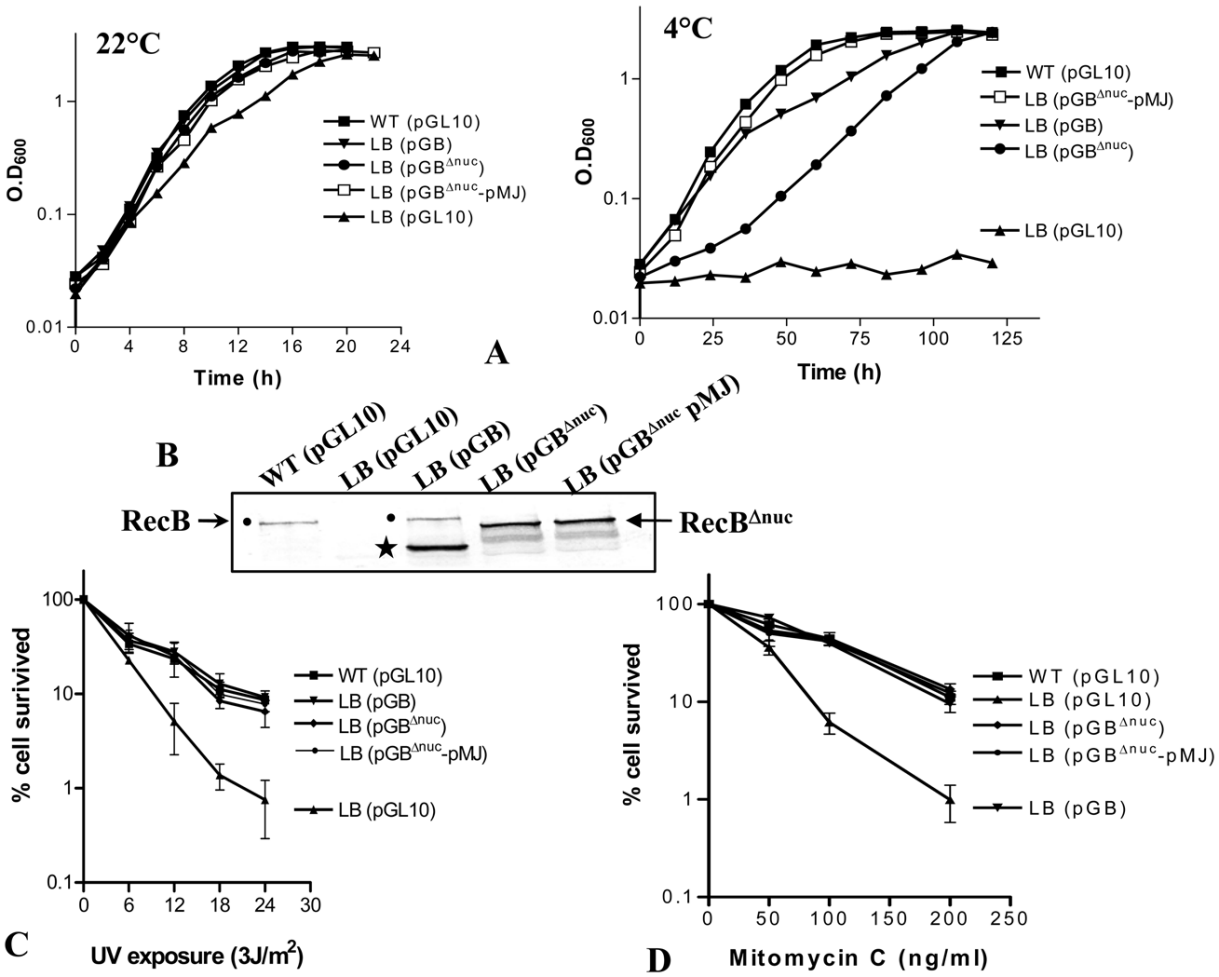
Importance of RecB nuclease-domain and RecJ exonuclease during growth, and in the repair of UV and mitomycin C induced DNA damage. The full-length RecB (from pGB), or the nuclease-deficient RecB^Δnuc^ (from pGB^Δnuc^), or both RecB^Δnuc^ and RecJ together (from pGB^Δnuc^ and pMJ, respectively) were expressed in the *ΔrecB* strain (LB). The strains were checked for growth at 22° and 4°C (**A**), and for the production of RecB peptides by Western analysis (**B**) using RecB-specific antibodies. The positions of full-length RecB and RecB^Δnuc^ peptides are marked by black dots (•) and arrow (←) respectively. Note that RecB degrades in cells when expressed in high amount from plasmid, which produce shorter peptides (marked by a ‘*’ in LB (pGB) lane). (**C**) and (**D**) show the UV and mitomycin C sensitivity of LB and LB complemented strains expressing the RecB and RecJ peptides from the indicated plasmids (shown within brackets).

The assessment of the importance of nuclease activity for repair of DNA and growth at low temperature was not quite obvious in the above experiment with *recB^D1118A^* allele of *P. syringae*. The chromosomal copy of the *recJ* gene, which encodes (5′→3′) RecJ-exonuclease, might complement the nuclease deficiency of the RecB^D1118A^CD enzyme *in vivo*, as seems to occur in *E. coli* [29]. Besides, the corresponding mutant RecB^D1080A^CD enzyme of *E. coli* was deficient not only in the nuclease activity but also in the RecA loading capacity [23], [25]. To address this issue, we constructed a plasmid pGB^Δnuc^ that produced a truncated RecB peptide lacking the most of nuclease domain (RecB^Δnuc^). We introduced pGB^Δnuc^ into LB (Δ*recB*) strain that produces RecC and RecD from the chromosome encoded genes. We then compared the growth and the DNA-damage sensitivity of LB expressing the full-length RecB or RecB^Δnuc^ peptides (Fig. 8). Both these peptides, produced from the multi-copy plasmids (pGB and pGB^Δnuc^), were able to complement the UV and MMC sensitivity of LB strain (Figs. 8C, 8D). However, only the full-length RecB could rescue the 4°C growth-defect of LB to the wild-type level; the RecB^Δnuc^ peptide complemented the defect only partially, leading to a slower growth rate (Fig. 8A). Upon expression of the RecJ exonuclease from another plasmid pMJ in the strain i.e., LB (pGRecB^Δnuc^), the growth rate was restored to the wild type level. The empty vector pMMB206 did not complement any of the defects of *ΔrecB* strain. This suggests that the RecB-borne nuclease activity of RecBCD complex *in vivo* is important but dispensable for growth of *P. syringae* at low temperature, possibly due to the activity of chromosomal *recJ* gene. A summary of all the findings on the *P. syringae* RecBCD enzymes are shown in Table 5.

**Table 5:** Summary of the *P. syringae recBCD* phenotypes.

P. syringae Enzyme	Activity in P. syringae				Activity assayed in E. coli				
	DNA damage-resistant to		Cell viability	Growth at 4°C	Recombination			ExoV Activity (T4 2^−^ growth inhibition)	λ Red^−^ Gam^−^ (both χ° and χ^+^) plaque size
	UV	MMC			Hfr	λ×λ	Chi		
RecBCD	+	+	+	+	+	+	–	+	large
RecBC	–	–	–	–	–	–	–	–	large
RecBD	–	–	–	–	nd	nd	nd	nd	nd
RecCD	–	–	–	–	nd	nd	nd	nd	nd
ReB^K28Q^CD	–	–	–	–	–	–	–	–^*^	large
RecB^D1118A^CD	+	+	+	+	–	–	–	–	large
RecBCD^K229Q^	–	–	–	–	–	–	–	–^*^	large
RecB^Δnuc^CD	+	+	nd	+^S^	nd	nd	nd	nd	nd
RecB^Δnuc^CD plus multi-copy RecJ	+	+	nd	+	nd	nd	nd	nd	nd

+^S^, slower growth; nd, not determined.

–^*^, 1000 times lower than ‘+’ value (1.0), but higher than ‘–’ values (E. O. P. ∼10^−6^).

### RecBCD and RecBC Enzymes of *E. coli* Are Fully Active in *P. syringae*

**Figure 9:**
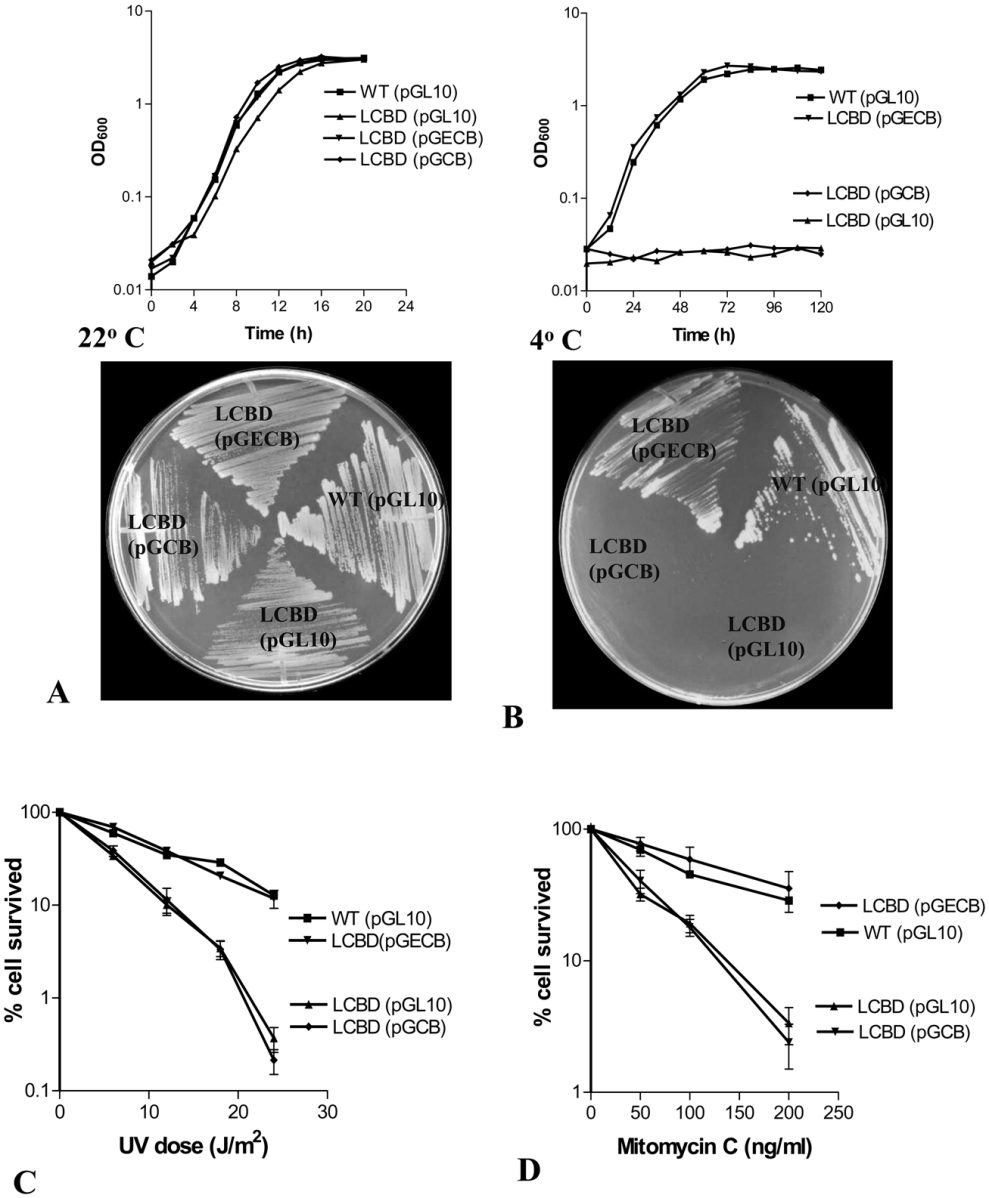
Complementation ability of dimeric RecBC enzymes. RecBC enzymes of *E. coli* (RecBC^Ec^) and *P. syringae* (RecBC^Ps^) were expressed from pGECB and pGCB plasmids respectively in LCBD (*ΔrecCBD*) strain of *P. syringae*. The mutants were then examined for growth at 22° (**A**) and 4°C (**B**), and for resistance to UV (**C**) and mitomycin C (**D**) following the methods described under Materials and Methods. Wild-type (WT) and LCBD strain harboring the empty plasmid vector pGL10 were used as the controls.

We earlier reported that *E. coli* RecD subunit individually failed to complement the low-temperature growth defect of *recD* inactivated strain CS1 [6]. To examine whether this is due to the cognate subunits-recognition problem we expressed all three subunits together from *E. coli*, i.e., whole RecBCD^Ec^, by expressing the genes from pGECBD plasmid in LCBD (*ΔrecCBD*) strain of *P. syringae*. The RecBCD^Ec^ enzyme indeed complemented the low temperature growth defect and the UV and MMC sensitivity of LCBD (Figs. 4A–D). To our surprise we also observed that RecBC enzyme of *E. coli* (RecBC^Ec^) lacking RecD subunit too is proficient, unlike the RecBC^Ps^ enzyme, in abrogating the defects of LCBD strain (Figs. 9A–D).

## Discussion

*P. syringae* Lz4W was isolated from the soil samples collected in and around Lake Zube (**Lz** stands for Lake Zube and **4W** stands for white colony no 4) of Schirmacher Oasis, Queen Maud Land (also referred to as Dakshin Gongotri Hill ranges by India) as a part of the study of microbial diversity in Antarctica [31]–[32]. The psychrotrophic bacterium has since been used as a model system for understanding the molecular basis of cold-adaptation [3]–[6], [33]. In this context, our finding that *recD* is required for growth of the bacterium at low temperature [6] led to the question whether the requirement is a function of the RecBCD complex, or the function of a yet unknown protein complex of RecD, or a novel function of RecD alone? To address this question, as well as to extend the issue further to find out the nature of RecBCD enzymatic activity that might be important for growth at low temperature, the present study was undertaken. The results unequivocally establish that all three subunits of the RecBCD complex are essential for growth at low temperature, and the RecD requirement in the Antarctic *P. syringae* is a function of the RecBCD enzyme. The data also suggest that the ATP-dependent activities of both motor proteins (RecB and RecD) are crucial for the RecBCD dependent functions in the organism.

The essentiality of RecB and RecC subunits in the DNA repair pathway is well established in *E. coli*, and therefore it is not so surprising that *P. syringae* lacking in any one of these two subunits are deficient in the recombination and repair of DNA, and that the inactivation of *recB* or *recC* leads to cell death and growth inhibition at low temperature. However, the observation that the lack of RecD subunit in the enzyme makes it impotent for DNA repair is significant, and appears to be unique to *P. syringae*. In the case of *E. coli*, the dimeric RecBC^Ec^ enzyme is not only proficient in homologous recombination and repair of DNA, the RecD subunit has been proposed to be anti-recombinogenic, as shown by the facts that the *recD*-deleted strains are hyper-recombinogenic, and RecBC enzyme loads constitutively the RecA protein on ssDNA for generating the recombination-intermediates [34]. More importantly, *E. coli recD* mutants do not affect cell survival or show any growth defect, suggesting its dispensability in the mesophile. Thus, the contribution of RecD subunit to the RecBCD activities in the psychrotrophic *P. syringae* appears to be fundamentally different from the mesophilic *E. coli*.

### Similarities between the RecBCD Enzymes of *P. syringae* and *E. coli*

By several criteria, such as recombination proficiency, nuclease activity inhibiting phage growth, and cell viability, the RecBCD^Ps^ enzyme is similar to RecBCD^Ec^, except that Chi-recombinational hot spots [35] have no detectable activity in the presence of RecBCD^Ps^ (Table 3). This result is consistent with the earlier observation that RecBCD enzymes from *Pseudomonas* group (*P. aeruginosa* and *P. putida*) do not recognize *E. coli* Chi sequence [36]. However, RecBCD^Ps^ enzyme appears to be half as efficient in the *E. coli* assay system, when compared with RecBCD^Ec^ activity for homologous recombination in both Hfr crosses and λ recombination assays (Table 3). On the other hand, both enzymes displayed similar and high level of the ExoV activity in the T4 2^-^ phage multiplication assay. Therefore, it is conceivable that the full complements of RecBCD enzyme, in spite of the difference in RecD requirement on the complex, are exchangeable between *P. syringae* and *E. coli*. However, it is significant that RecBCD^Ec^ enzyme can complement the growth defects of LCBD (*ΔrecBCD*) strain of *P. syringae* at 4°C, a temperature in which *E. coli* does not grow. This suggests that the mesophilic RecBCD^Ec^ enzyme is functionally active even at low temperature. An evolutionary implication of this, taking the fact that RecBCD^Ps^ is also functional at 37°C in *E. coli*, would be that the ancestors of the two bacterial species belonging to Pseudomonadaceae and Enterobacteriaceae family of γ-Proteobacteria might have had a wider range of growth temperatures.

The reciprocal ability of RecBCD^Ps^ and RecBCD^Ec^ to complement the defects of *ΔrecBCD* strains of *P. syringae* and *E. coli* suggests that the enzyme complex as a whole have retained all conserved biochemical activities that are needed for DNA repair and recombination. On the other hand, we have observed that *ΔrecBCD* strains expressing the complementary subunits from the two species, e.g., RecBC^Ps^ and RecD^Ec^, or RecBD^Ps^ and RecC^Ec^, or RecCD^Ps^ and RecB^Ec^ generating possibly the chimeric enzymes of RecBCD in the cells are neither proficient in DNA repair, nor have the ability to support growth of *P. syringae* at 4°C (data not shown). This suggests that cognate recognition of the subunits is essential for constituting a functionally active RecBCD enzyme, as RecB, RecC, and RecD sequences of *P. syringae* Lz4W and *E. coli* are only 39, 41, and 43% identical (53, 57, and 56% similar) between them, respectively. However, the ability of RecBC^Ec^ enzyme (lacking RecD), unlike the RecBC^Ps^, to complement the defects of LCBD strain was a little surprising. The fact that both RecBC^Ps^ and RecBC^Ec^ enzymes are lacking in exonuclease activity, as evidenced by the T4 2^-^ phage multiplication assay, it is likely that the unique necessity of RecD in the RecBCD^Ps^ might not be due to the lack of nuclease activity on the enzyme.

Nuclease activity was shown to be essential for *recBCD*-dependent pathway of recombination and repair in *E. coli* [30]. However, in the absence of exonuclease (ExoV) activity of RecBCD, *E. coli* depends on other ss-DNA exonucleases for homologous recombination in cells [37], [38]. In the case of *P. syringae*, LCBD expressing the RecB-nuclease deficient enzymes (e.g., RecB^D1118A^CD and RecB^Δnuc^CD) show proficiency in DNA repair, suggesting that the activity has been compensated *in vivo* by other exonucleases in the cells. Our study indicates that RecJ exonuclease encoded by the *recJ* gene on chromosome is one such candidate. The evidence comes from our observation that the growth rate of slow-growing LB is enhanced to the wild-type level, by expressing RecB^ΔNuc^ and RecJ polypeptides simultaneously in cells. In this respect, the situation is similar to *E. coli*, where the *recJ*-dependent hybrid pathway of recombination operates when there is a defect in the *recBCD* pathway [39].

### Importance of the Degradation Versus DNA Unwinding Functions of RecBCD Enzyme at Low Temperature

For an effective response to cellular damages, cells in general employ alternative mechanisms: one that can tolerate and/or repair the assaults to keep alive, and the other that degrades to eliminate the irreparable molecules which on accumulation might even lead to cell death. Both mechanisms appear to be operating in *P. syringae* at low temperature, as the risks of DNA damage become higher affecting viability and growth. At low temperature, DNA damage is likely to increase for two main different reasons: (i) level of intracellular reactive oxygen species is enhanced due to lower respiration rate, causing the damage [40], [41] and as a consequence, (ii) frequent collapsing of replication forks (RFs) occurs in dividing cells due to the stalling of replication machinery at the damaged RF [16]. Although bacterial cells have evolved elaborate DNA repair machinery which is sufficient under normal circumstances, they fail when the damages become too many, or are of irreparable type. The degrading activity of the DNA repair machinery is probably needed to destroy the irreparable RFs to protect the cells, in the absence of which damaged DNA would accumulate in the cells. Interestingly, the RecBCD nuclease activity is additionally employed in bacteria for protecting the cells from foreign DNA invasion and bacteriophage infection/multiplication [11]. Our observation that short DNA fragments accumulate at 4°C in the mutant *P. syringae* cells due to inactivation of the genes in *recCBD* operon, suggests that these DNA fragments would otherwise be cleared by RecBCD-dependent nuclease activity in wild-type cells.

The evidence that the load of DNA damage is very high in *P. syringae* at low temperature comes from the observation that, even in wild-type cells, the amount of linear chromosomal DNA is higher at 4°C than at 22°C (Fig. 3D and Supporting Information S1). The wild-type cells however exhibit very little accumulation of shorter DNA fragments, which are generally visible prominently as smear in the ∼30–50 kbp region of the gels. In contrast, *recBCD* defective cells shows higher amount of both linear chromosomal DNA and shorter DNA fragments. This suggests that the defects in these cells probably lie not only in the repair, but also in the degradation of the irreparable DNA, leading to the accumulation of shorter DNA fragment-smears. Although the nature and source of these DNA smears have not been addressed in this work, two kinds of circumstantial evidence suggest that the collapsed replication forks (RFs) might be a source. Firstly, we have noticed that the cells with defects in the RecBCD enzyme die faster at 4°C under growing conditions (e.g., in ABM) than under non-growing (e.g., in minimal growth medium lacking carbon source) conditions (unpublished observation). Secondly, the nuclease deficient LB (*ΔrecB*) cells expressing RecB^Δnuc^ peptide display proficiency in DNA repair as shown by the resistance to UV and MMC to the wild-type level, but grow slowly at 4°C; in the latter case most cells are likely to contain RFs on the replicating chromosomes. This result can also be interpreted as an indication that the repair of replicating DNA is more dependent on the RecBCD associated nuclease than probably on the RecJ nuclease *in vivo*, since RecJ protein is present in cells under both conditions. An alternative explanation that the *recJ* expression at 4°C is very poor could not be ruled out in this study. Nonetheless, it is important to note that the degradation of DNA by RecBCD also requires unwinding of DNA duplex strands, which is provided by the helicase activities of RecB and RecD subunits. The helicase activity of RecBCD is all the more crucial in the case of cold-adapted *P. syringae*, due to the low temperature induced higher stability of duplex DNA structures that would require additional higher processivity and stronger helicase activity of the enzyme. This could be the reason why helicase activity of RecBCD^Ps^ is dependent on both RecB and RecD subunits.

In addition to the helicase and nuclease activities, the ability of RecBCD to load RecA on ssDNA for initiation of strand transfer reaction and pairing with homologous DNA sequence is an important property of the enzyme. The C-terminal nuclease domain of RecB subunit (RecB^nuc^) is involved in the RecA loading [42]. Although Chi sequence plays a major role in selection of the recombination site in *E. coli*, probably due to the RecA loading capacity of Chi-modified RecBCD enzyme, this *cis*-element is not an absolute requirement for homologous recombination. The RecBC enzyme of *E. coli* can stimulate recombination independent of Chi, due to the constitutive RecA-loading property of the enzyme in absence of the inhibitory RecD subunit. In the case of *P. syringae*, RecBCD lacking RecD becomes functionally inactive, and hence the inhibitory activity of RecD^Ps^ for RecA loading on ssDNA does not arise. Additionally, the observation that LB (*ΔrecB*) strain expressing RecB^Δnuc^CD enzyme, which lacks the one and all nuclease and RecA-loading domain of RecB, is proficient in the recombinogenic repair of UV-damaged DNA suggests that the mutant enzyme is probably dependent on the RecFOR function for RecA loading. In this respect the *E. coli* RecB^Δnuc^CD enzyme was surprisingly different, which failed to restore recombination proficiency and UV resistance of *ΔrecB* cells [43].

### Requirement for the Synergy of RecB and RecD helicase Motor Functions in *P. syringae*

Both RecB and RecD subunits in the trimeric RecBCD complex have DNA motor helicase activity, powered by the hydrolysis of ATP. These two proteins can independently translocate as the monomeric motors, along the anti-parallel strands (3′→5′ and 5′→3′) of DNA, as observed in the *in vitro* assays [44], [45]. However, how these two autonomous motors on the RecBCD complex are regulated during the translocation and unwinding of DNA are not properly understood. Two general models involving ‘uncoupled translocation’ and ‘concerted translocation’ of the motor subunits have been considered to explain the change of velocity of the two motor proteins in response to the regulatory ‘Chi’ sequences in the case of *E. coli*. Independent methods, such as EM analysis of DNA-unwinding intermediates forming ssDNA loop-tail prior to Chi [44], [45] and the optical trap method of single molecular tracking of ssDNA-RecBCD complex [46], have confirmed that the translocation activity of the two motors are ‘uncoupled’ to each other before encountering Chi. RecD acts as the ‘fast’ or lead motor before Chi, while RecB becomes the lead motor only after the Chi. Although how the asynchronous speeds of RecB and RecD are coordinated in the complex is unknown, the ‘intersubunit signaling cascade’ that has been recently proposed for the cleavage site determination on DNA by RecBCD [47] might be a key factor. The ‘uncoupled translocation’ of the motor units in principle can give flexibility to the RecBCD enzyme to work even when one of the motors is inactivated (e.g., in RecBCD^K229Q^); however, the maintenance of autonomy of the dual motors, from adaptive point of view, might not be helpful when synergy and efficiency are needed under conditions that influence thermal stability of DNA secondary structures. In the Antarctic *P. syringae*, low temperature stabilized DNA secondary structures would require the synergy between the two helicase motors in RecBCD. The concerted translocation of both RecB and RecD motors might be so important that the defect in motor activity of any one of them makes the RecBCD^Ps^ inefficient, leading to the failure of the enzyme to function at low temperature. Although this does not explain the sufficiency of RecBC^Ec^ in overcoming the problems at low temperature, it is possible that the RecBCD machineries in Enterobacteriaceae and Pseudomonaceae have evolved independently, leading to the difference not only in the inter-subunit dependency but also in the efficiency of the motor proteins in the two bacterial species. In this context, the findings of Spies *et al* [48] that the inactivation of RecD motor in *E. coli* enzyme (RecBCD^K177Q^) keeps the enzyme complex almost fully functional, unlike the RecBCD^K229Q^ of *P. syringae*, is important and supports the above hypothesis. Our own observation, which shows that the RecD subunit adds to the overall stability to the RecBCD^Ps^ complex in *P. syringae* (Pavankumar TL and Ray MK, unpublished observation) also supports that the subunits of RecBCD complex have coevolved but independently in different groups keeping overall role of the enzyme unchanged in bacterial cells.

In conclusion, our study shows that all three subunits of the RecBCD enzyme are essential for physiological activities of the enzyme in the Antarctic *P. syringae*, namely, repairing of DNA damage and supporting the growth at low temperature. The RecBCD enzymes are exchangeable between the psychrophilic *P. syringae* and the mesophilic *E. coli* when provided with the entire protein complex from same species. However, the RecBC proteins (RecBC^Ps^ and RecBC^Ec^) of the two bacteria are not equivalent; the RecBC^Ec^ is proficient in DNA recombination and repair, and supports the growth of *P. syringae* at low temperature, while RecBC^Ps^ is insufficient for these functions. Finally, both helicase and nuclease activity of the RecBCD^Ps^ are although important for DNA repair and growth of *P. syringae* at low temperature, the RecB-nuclease activity is not essential *in vivo*.

## Materials and Methods

### Bacterial and Phage Strains, Plasmids, and Growth Conditions

Bacteria and bacteriophages, and plasmids used in this study are described in Table 1 and 2 respectively. *P. syringae* Lz4W was grown routinely in Antarctic Bacterial Medium (ABM) (5 g liter^−1^ peptone and 2.5 g liter^−1^ Yeast extract) at 22° or 4°C as described [6]. *E. coli* cells were grown at 37°C in LB medium [49]. When required, culture media were supplemented with antibiotics at the following concentrations: ampicillin, 100 µg ml^−1^; tetracycline, 20 µg ml^−1^; kanamycin, 50 µg ml^−1^ and gentamycin, 10 µg ml^−1^. For determination of generation time and growth analysis, all experiments were performed with exponentially grown cells. Generally, *P. syringae* cultures (∼0.6 OD_600_) were freshly inoculated into ABM broth at a 1% dilution and incubated at 22° and 4°C with aeration by shaking; optical density of the cultures at 600 nm (OD_600_) was measured at different time intervals. Generation times were calculated from the growth curves of the strains in ABM. The cfu (colony forming units) measurements were performed by plating the cultures on ABM-agar plates with appropriate serial dilutions.

### General Recombinant DNA Method

General molecular biology techniques including isolation of genomic DNA, polymerase chain reactions (PCR), restriction enzyme digestion and ligation, electroporation, Southern hybridization, Western analysis, etc were performed as described earlier [49]. All restriction enzymes, T4 DNA ligase, T4 polynucleotide kinase, and other enzymes used in this study were from New England Biolabs (NEB, Ipswich, MA, USA). pMOS*Blue* blunt end cloning kit was from Amersham Biosciences (Uppsala, Sweden). Polymerase chain reactions were carried out using proof reading pfx DNA polymerase from Invitrogen (San Diego, CA, USA). PCR products were purified by Qiagen PCR purification kit (Qiagen, Hilden, Germany). DNA Sequencing reactions were carried out using ABI PRISM Dye terminator cycle sequencing method (Perkin-Elmer, Boston, USA) on an automated DNA sequencer (ABI model 377). Oligonucleotides were purchased from a commercial source (Bioserve Biotechnology, Hyderabad, India).

### Generation of *recC, recB, recD*, and *recCBD* Null Mutants of *P. syringae*

The common strategy adopted for gene/s disruption was to insert a tetracycline resistance gene (*tet^R^*) cassette (Tc-cassette) by replacing a middle portion of the target gene/s. Adequate length of homologous DNA sequence was provided on either side of the Tc-cassette for double crossover recombination to occur between the suicidal plasmid constructs and *P. syringae* chromosome. We constructed four suicidal plasmid vectors, pJQC^tet^, PJQB^tet^, pJQD^tet^ and pJQCBD^tet^ for disruption of the *recC, recB, recD* individually, and for deletion of the whole *recCBD* operon. Each plasmid construct contained in the multiple cloning site (MCS) of pJQ200SK [50] a Tc-cassette flanked by the 5′ and 3′ DNA regions of target gene(s). Briefly, the descriptions of the constructs are as follows. (i) pJQC^tet^: DNA segment containing *recC* 5′end (890 bp) - Tc-cassette – *recC* 3′end sequence (663 bp) cloned into *Xba*I and *Sal*I sites of pJQ200SK; (ii) pJQB^tet^: 754 bp *recB* 5′end sequence- Tc-cassette – *recB* 3′end (1,535 bp) cloned into *Bam*HI site of pJQ200SK; (iii) pJQD^tet^: 670 bp *recD* 5′end - Tc-cassette- *recD* 3′end (1000 bp) cloned into *Xba*I and *Sal*I sites of pJQ200SK; (iv) pJQCBD^tet^: 5′end of *recC* (890 bp DNA)- Tc-cassette - 3′end of *recD* (1000 bp) cloned into *Xba*I and *Sal*I sites of pJQ200SK. The Tc-cassette (∼2.5 kbp) was taken out as *Pst*1 fragment from pMOS^tet^, which was constructed by cloning the tetracycline resistance gene of pTc28 [6] into the pMOS*Blue* vector.

Gene disruption by homologous recombination method is not efficient in *P. syringae*. We therefore introduce alkaline denatured DNA into cells by electroporation, which enhances the frequency of homologous recombination in *P. syringae*. Alkaline denaturation of pJQC^tet^, pJQB^tet^, pJQD^tet^, and pJQCBD^tet^ was performed as described earlier [51], and the electroporation was carried out using a gene pulser II (BioRad, Harcules, CA). The electroporated cells were plated onto tetracycline containing ABM-agar plates. The tetracycline-resistant transformants were screened by PCR using appropriate gene specific primers, for checking the replacement of wild-type genes on chromosome. The deletion-insertion alleles of *recC, recB, recD,* and *recCBD* on chromosome were further confirmed by Southern analysis, in which the genomic DNAs were digested with *Pst*I or *Nco*I restriction enzymes, separated on 1% agarose gel, and then blotted onto the Hybond N^+^ membrane (Amersham biosciences). Full-length *recC, recB, recD,* and *recCBD* gene/s were used as ^32^P-labeled probes for Southern hybridization, and the radioactive signals were detected and analyzed by a phosphorimager (Fuji FLA-3000). The mutant *ΔrecC*, *ΔrecB, ΔrecD,* and *ΔrecCBD* strains were named as LC, LB, LD and LCBD respectively.

### Construction of Plasmid Vectors for Expression of Genes and Complementation Studies

For genetic complementation analysis, plasmids were constructed for the expression of *recB*, *recC* or *recD* individually or in combinations, using the broad-host-range plasmid pGL10 as described earlier [52]. The primers used for PCR amplification of the genes are enlisted in Supporting Information S1. Briefly, for the construction of pGC which expressed RecC, 3.45 kbp DNA of *recC* open reading frame (ORF) was first amplified by the FCN1 and RCS1 primers using the *P. syringae* genomic DNA as template. The gel-purified PCR product was digested with *Nde*I and *Sac*I and ligated in-frame with the N-terminal 6×His-tag sequence of the pET28b expression vector, generating pETC. Then, the DNA fragment containing His-tagged *recC*-ORF was released from pETC by digesting the plasmid with *Xba*I and *Sac*I, and ligated into pGL10 to generate pGC. Similarly, for the construction of pGB^His^ that expressed RecB, 3.684 kbp *recB* gene was amplified using the BPF and DPE primers, and initially cloned into pMOS*Blue* blunt end vector (Amersham Biosciences). Subsequently, the *recB* was released by digesting with *Bam*HI and ligated in-frame with the N-terminal 6×His-tag coding sequence of pET28b vector to generate pETB. The His-tagged ORF of *recB* gene was then cleaved out from pETB as (*Xba*I-*Sac*I) fragment and ligated into pGL10 to generate pGB. The pGD, which produced C-terminally 6×His-tagged RecD, has been described earlier [52]. For the construction of pGCBD which expressed all three subunits of RecBCD^Ps^, 9.215 kbp DNA containing the three reading frames of *recCBD* operon of *P. syringae* was amplified by PCR using FCNH1 and RDS1 primers. The purified PCR product was then digested with *Nhe*I and *Sac*I and ligated to in-frame with the translation start site of pET28b expression vector. Then, *recCBD* with the in-frame His-tag coding sequence of the vector was released by digesting with *Xba*I and *Sac*I, and ligated into pGL10, generating pGCBD. This plasmid construct produced RecC as the only N-terminally His-tagged protein, while the RecB and RecD were not tagged. The pGECBD plasmid was constructed to produce *E. coli* RecBCD proteins in *P. syringae*. For the construct, 18.5 kbp of *E. coli* chromosomal DNA containing *recC*, *recB* and *recD* genes between the *thy*A to *arg* A was released from pFS-11-04 [53] by *Bam*HI digestion, and ligated into the *Bam*HI site of pGL10. Similarly, for the construction of pGECB that produced dimeric RecBC^Ec^ protein, 11.7 kbp DNA containing the *recC*-*ptr*-*recB* region of *E. coli* was cleaved out from the plasmid pAMP3 [54] as *Bam*H1 fragment and then cloned into the *Bam*H1 site of pGL10.

### Site-Directed Mutagenesis and Generation of *recB^K28Q^*, *recB^D1118A^*, and *recD^K229Q^* Alleles

Site-directed mutagenesis reactions were performed using ‘QuickChange XL kit’ (Stratagene, USA) as per the manufacturer’s guide lines. Mutations were created directly in the *recCBD* operon on pGCBD plasmid that expressed all three subunits of the RecBCD^Ps^ enzyme. The *recB^K28Q^*, and *recB^D1118A^* alleles were created by substituting the respective codons of lysine (AAA) and aspartic acid (GAC) residue with the codons for glutamine (CAA) and alanine (GCC) at the residue positions 28 and 1118 of RecB-ORF. The *recD^K229Q^* allele was created by replacing the codon of lysine (AAA) with glutamine (CAA) at the 229 residue position of RecD-ORF. The mutagenic primer sets employed for substitutions of the codons in *recB* and *recD* genes are enlisted in Supporting Information S1. All mutations were confirmed by DNA sequence analysis, and the proteins expression was confirmed by Western analysis.

### Cloning and Expression of RecJ and RecB^Δnuc^ in *P. syringae*

*recJ* ORF was amplified from genomic DNA using the primer sets (JFN1 and JRE1) and cloned initially in *Nde*I and *Eco*RI sites of pET28b for expression in *E. coli*, and subsequently in the *Sma*I cloning site of the broad-host-range plasmid pMMB206 (IncQ *ori*) to generate pMJ that can coexist with pGL10 (IncP *ori*) derivatives in *P. syringae* cells. For the production of RecB^Δnuc^ peptide, pGB was digested with *Eco*RI and religated to produce pGB^Δnuc^. This construct produced truncated RecB peptide (1–1062 amino acids long) lacking the C-terminal 165 amino acids from the nuclease domain of RecB (full length 1227 amino acids).

### Genetic Complementation Analysis

Complementation analyses were carried out by mobilizing the pGL10-based plasmid constructs containing the relevant gene/s (see above) into the *ΔrecC*, *ΔrecB*, *ΔrecD* and *ΔrecCBD* strains of *P. syringae*. Briefly, bi-parental conjugation was set up between the donor *E. coli* S17-1 strain [55] harboring the pGL10 derivatives (pGC, pGB, pGD, pGCBD, pGECBD, pGECB, and others) and the recipient strains (LC, LB, LD and LCBD). Transformed mutants were selected on ABM-agar plates containing tetracycline and kanamycin at 22°C. Expression of the plasmid-borne genes in *P. syringae* mutants were confirmed by Western analysis using RecC, RecB, and RecD specific rabbit polyclonal antibodies, which were raised in the laboratory.

### Recombination Assays and Chi (χ) Activity Measurement

Recombination frequency was measured in *E. coli* Hfr (high-frequency recombination) conjugation experiments and in λ phage crosses as described [22], [25]. *E. coli* recipient strains V67 (*recB21::IS186 his^−^ Str^R^* F^−^) or V66 (isogenic *recBCD^+^*) harboring the plasmid borne *recCBD* alleles or the empty plasmid vector alone were crossed with the donor *E. coli* strain V1306 (Hfr PO44 *his^+^ Str^S^*). The ratio of Hfr:F^−^ cells in the matings was about 1:10. The number of *his^+^*(*Str^R^*) recombinants per Hfr donor cell, corrected to the viability of recipient, was calculated for measuring recombination proficiency.

The phage strains (Table 2) used in the study was received from Gerald Smith's laboratory (Fred Hutchinson Cancer research Center, Seattle, USA). The frequency of J^+^R^+^ recombinants in the mixed λ phage crosses (phage 1081×1082 and phage 1083×1084) was determined by plating them on *E. coli* strain 594 (*sup^+^*) for recombinants and on strain C600 (*supE*) for total phage titer. The Chi activity in these crosses was determined by the method of Stahl and Stahl [35] using the equation, 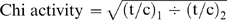, where (t/c) is the ratio of turbid to clear plaques from cross 1 or cross 2, among J^+^R^+^ recombinants as described [25]. Additionally, phage plaque size tests using λ Red^−^ Gam^−^ phages lacking Chi (χ^o^) (strain 872) or with Chi (χ^+^) (strain 873) on the lawns of *E. coli* cells were performed as described previously [22].

### T4 and T4 2^-^ Phage Multiplication Assay for Exonuclease Activity

*In vivo* exonuclease activity of the *recBCD* alleles were determined by T4 and T4 2^-^ phage multiplication assays as described [22]. *E. coli* V330 and V66 strains were transformed with the pGL10 derived constructs expressing different *recBCD* alleles for the phage assays.

### Cell Viability Test and Microscopic Analysis

Cell viability was determined by plating the exponentially growing cells, with appropriate dilution, onto ABM-agar plates and counting the colonies on the plates. For this, overnight grown cells were freshly inoculated in ABM broth and incubated at 22°C with aeration till the culture density reaches ∼0.5 OD_600_, when the cells are diluted and plated. The relative cell viability was calculated as the number of cells (cfu) per ml divided by the OD_600_ of that culture, and normalized by the value of wild-type strain of *P. syringae*, which generally gave ∼4.3×10^7^ cfu/ml at an OD_600_ value of 0.5 measured in a 1 cm cuvette. For measuring the cell viability at low temperature, the 22°C grown cultures were shifted to 4°C and at every 24 hrs of intervals cells were plated onto ABM-agar plate with appropriate dilutions. Plates were incubated at 22°C for 48 hrs before counting the cfu on plates. Percentage viability of each strain was calculated by considering their respective cfu at 0 hr (just before shifting from 22° to 4°C) as 100%. For microscopic analysis of cell viability, cells were stained with the LIVE/DEAD BacLight viability kit (Molecular Probes) and examined under fluorescence microscope (Carl Zeiss, Germany) as described [6]. Cell size was measured from the phase contrast images using a axiovision version 3.1 software provided with the Zeiss microscope (Axioplan imaging).

### UV and Mitomycin C (MMC) Sensitivity Tests

Sensitivity to UV and MMC was tested as described earlier [6]. Briefly, *P. syringae* cells on ABM-agar plates were exposed to UV light at a dose rate of 3 J/m^2^/sec, and incubated at 22°C in the dark for 48 hrs. Survivors of each strain were counted as colonies (cfu) on plates, and the percentage of survival was calculated by considering the colony numbers on unirradiated plates as 100%. For MMC sensitivity tests, cells (∼0.5 OD_600_) were incubated for 30 min in cultures with different concentration of MMC, washed and serially diluted with fresh ABM, and then spread onto ABM-agar plates. Following incubation at 22°C for 48 hrs, the percentage survivors on plates were calculated by considering the cfu values of untreated cells as 100%.

### Raising Antibodies and Western Analysis

Polyclonal antibodies were raised against the His-tagged RecC and RecB proteins of *P. syringae* in rabbits using standard protocol [49]. Production of anti-RecD antibodies has been described [52] and the anti-His antibodies were bought commercially (Santa Cruz Biotechnology). For Western analysis, proteins were separated by SDS-PAGE, transferred onto Hybond-C membrane (Amersham Biosciences) and probed with RecB-, RecC-, RecD-specific antibodies or anti-His antibodies. The immune-reactive protein bands were detected by alkaline phosphatase conjugated anti-rabbit goat-antibodies (Bangalore Genie, India).

### Pulsed Field Gel Electrophoresis (PFGE)

PFGE was performed as described earlier [6] with minor modifications in the gel-running conditions. Typically, cell samples were harvested from the cultures of exponential phase (OD_600_ ∼0.5), and embedded in agar blocks (1% LGT agarose, FMC-Bioproducts, Rockland, ME). Each agarose block contained ∼0.5×10^7^−10^8^ cells. Electrophoresis was performed using CHEF-DRII (Bio-Rad) at a constant voltage (120 V) and with increasing pulse time of 60–120 seconds over a period of 24 hr at 14°C. For molecular size markers, concatemers of λ phage DNA and yeast chromosomes (NEB) were used.

## Supporting Information

Supporting Information S1 This file contains two tables (Tables ST1 and ST2) and three figures (Figures S1, S2, and S3). Table ST1 contains the cell size measurement data of *recBCD* mutants of *P. syringae*. Table ST2 contains the list of oligonucleotide primers and their sequence. Figure S1 depicts the LIVE/DEAD staining of the wild-type and *recBCD* mutant cells of *P. syringae*. Figure S2 shows the ability of *E. coli* RecBCD enzyme and the *P. syringae* RecBCD enzyme subunits to complement the cell survival defects and the damaged DNA accumulation defects of the LCBD (*ΔrecCBD*) strain. Figure S3 shows the amino sequence alignments of the ATP-binding site, the nuclease catalytic site of the RecB subunit, and the ATP-binding site of RecD to depict the conservation of the sites, and the residues that were mutated in the subunits.
(0.30 MB PDF)
