# Supplementary material for: All Three Subunits of RecBCD Enzyme Are Essential for DNA Repair and Low-Temperature Growth in the Antarctic Pseudomonas syringae Lz4W
Source: PLoS One. 2010 Feb 25;5(2):e9412. doi: 10.1371/journal.pone.0009412 (PMC2828478; doi:10.1371/journal.pone.0009412)
Supplement: Supporting Information S1 — This file contains two tables (Tables ST1 and ST2) and three figures (Figures S1, S2, and S3). Table ST1 contains the cell size measurement data of recBCD mutants of P. syringae. Table ST2 contains the list of oligonucleotide primers and their sequence. Figure S1 depicts the LIVE/DEAD staining of the wild-type and recBCD mutant cells of P. syringae. Figure S2 shows the ability of E. coli RecBCD enzyme and the P. syringae RecBCD enzyme subunits to complement the cell survival defects and the damaged DNA accumulation defects of the LCBD (ΔrecCBD) strain. Figure S3 shows the amino sequence alignments of the ATP-binding site, the nuclease catalytic site of the RecB subunit, and the ATP-binding site of RecD to depict the conservation of the sites, and the residues that were mutated in the subunits. (0.30 MB PDF) [file pone.0009412.s001.pdf]

SUPPLEMENTARY INFORMATION

"All three subunits of RecBCD enzyme are essential for DNA repair and low-temperature growth in the Antarctic *Pseudomonas syringae* Lz4W"

Authors: **Pavankumar T. L., A. K. Sinha, and M. K. Ray**

Supplementary* TABLE *ST1. Increased cell size of *recCBD* mutants of *P. syringae* at low temperature (4°C). Cell size was measured as described under Materials and Methods.

Strain	Cell size at 22°C (pre-shift) (length × width) μm	Cell size at 4°C (96 hr post shift) (length × width) μm
Lz4W (WT)	2.68 ± 0.44, 1.01 ± 0.22	1.42 ± 0.21, 0.74 ± 0.91
LC (*ΔrecC*)	3.34 ± 0.54, 1.12 ± 0.13	4.50 ± 0.92, 1.10 ± 0.16
LB (*ΔrecB*)	3.63 ± 0.60, 1.08 ± 0.18	4.37 ± 1.06, 1.2 ± 0.25
LD (*ΔrecD*)	3.59 ± 0.61, 1.22 ± 0.13	4.61 ± 0.91, 1.31 ± 0.26
LCBD (*ΔrecCBD*)	3.60 ± 0.59, 1.18 ± 0.13	4.81 ± 1.11, 1.14 ± 0.25

Supplementary* TABLE *ST2. List of primers used in this study for cloning and mutagenesis of *recCBD* genes of *P. syringae.* The restriction enzyme recognition sites are underlined, and mutated codons are shown in bold in the mutagenic primers.

Primer	Sequence (5'- 3')	Related information
FCN1	GGAATTCCATATGCCGGTTGCTACGTCC	*recC* forward primer with *Nde*I site
FCNH1	GCCTAGCTAGCATGCCGGTTGCTACGTCC	*recC* forward primer with *Nhe*I site
RCS1	CTTGCGAGCTCATTAGCGGGACGCCTCGC	*recC* reverse primer with *Sac*I site
BPF	CGGGATCCAATGAACGAGCAAAAAACACC	*recB* forward primer with *Bam*HI site
DPE	CAGCTAGGCAAATGAACGACTCAT	*recB* reverse primer with stop codon
RDS1	CTGTCGAGCTCCTTCAGCCCCAGCATCAAG	*recD* reverse primer with *Sac*I site
CDP1	CGCAAAACTGCAGCAAGACCTCGACGG	Forward primer with *Pst*I site for *recD* 3'end region amplification
JFN1<br>JRE1	AAGCTTAGCCATATGCCCACCGTACCTCGCCGCTTG<br>TCTACTAGTGAATTCTCACACGGTCTCATTGCCGCG	Forward and reverse primers set for *recJ* amplification
FBK28Q<br>RBK28Q	GCGCGGGCACCGGC**CAA**ACCTTCACCATC<br>GATGGTGAAGGT**TTG**GCCGGTGCCCGCGC	Forward and reverse primers set for creating *recB*^{K28Q} point mutation pGCBD
FBD1118A<br>RBD1118A	CGTTACTACGTGGCG**GCC**TACAAATCCAAC<br>GTTGGATTTGTA**GGC**CGCCACGTAGTAACG	Forward and reverse primers set for creating *recB*^{D1118A} point mutation in pGCBD
FDK229Q<br>RDK229Q	GGTCCCGGTACGGGT**CAA**ACCACCACGGTG<br>CACCGTGGTGGT**TTG**ACCCGTACCGGGACC	Forward and reverse primers set for creating *recD*^{K229Q} point mutation in pGCBD

Legends to the Supplementary Figures

Figure S1. LIVE/DEAD staining of *P. syringae* cells after the shifting of cultures from 22° to 4°C. The cells were stained with LIVE/DEAD Baclight bacterial viability kit (Molecular Probes, Eugene, OR) that comprises fluorescence staining dyes Syto9 and propidium iodide (PI). Syto9 stains live cells green, and PI stains dead cells red. The representative panels show the fluorescent labeled cells after 96 hours of the shift to 4°C. Note that the mutant cells of LC, LB, LD, and LCBD become longer and larger, while the wild-type cells (WT) become smaller and roundish at the low temperature (4°C).

Figure S2. *E. coli* RecBCD enzymes produced from the plasmid complement the cell survival defects (**A**), and the damaged DNA accumulation in LCBD strain *(ΔrecCBD*) of *P. syringae* (**B**) at low temperature (4°C). Cell survival was tested by measuring the cell numbers (cfu) at different time points, following the shift of cultures of the indicated strains from 22° to 4°C. The mutants harboring the respective plasmids have been indicated.

Figure S3. Amino acid sequence alignment of the conserved ATP-binding **(A)**, and nuclease catalytic **(B)** sites of RecB, and the ATP binding site RecD **(C)** subunits to indicate the conserved nature of the amino acid residues of the sites, and the mutations that were created in the conserved residues of the respective subunits of the *P. syringae* RecBCD enzymes (RecB^{K28Q}CD, RecB^{D1118A}CD and RecBCD^{K229Q}). Corresponding E. coli mutations of the RecB and RecD subunits have also been indicated. Sequence alignments were performed using the Clustal W programme, available at the NCBI site (www.ncbi.nlm.nih.com).

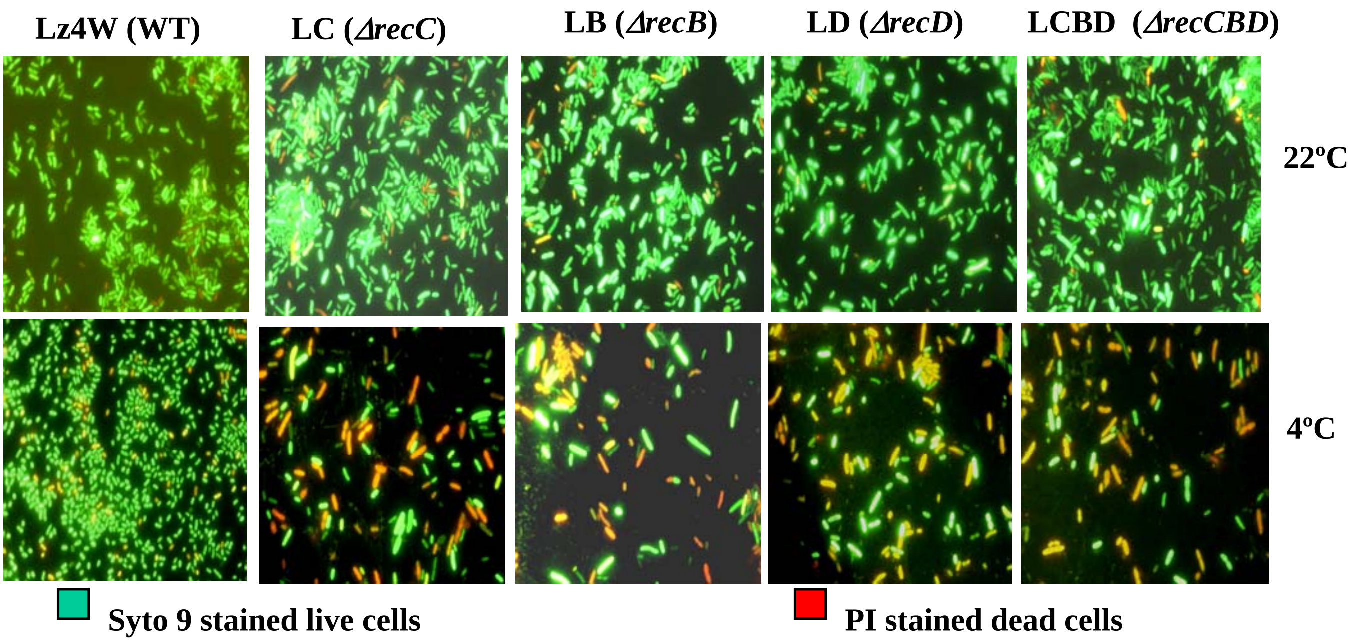

Supplementary **Figure** ***S1***

A

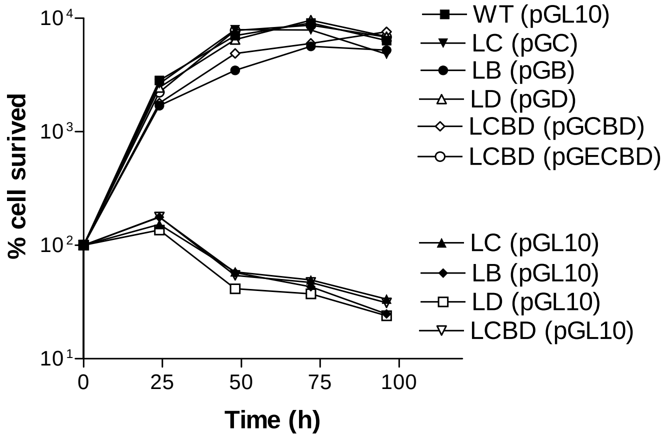

B

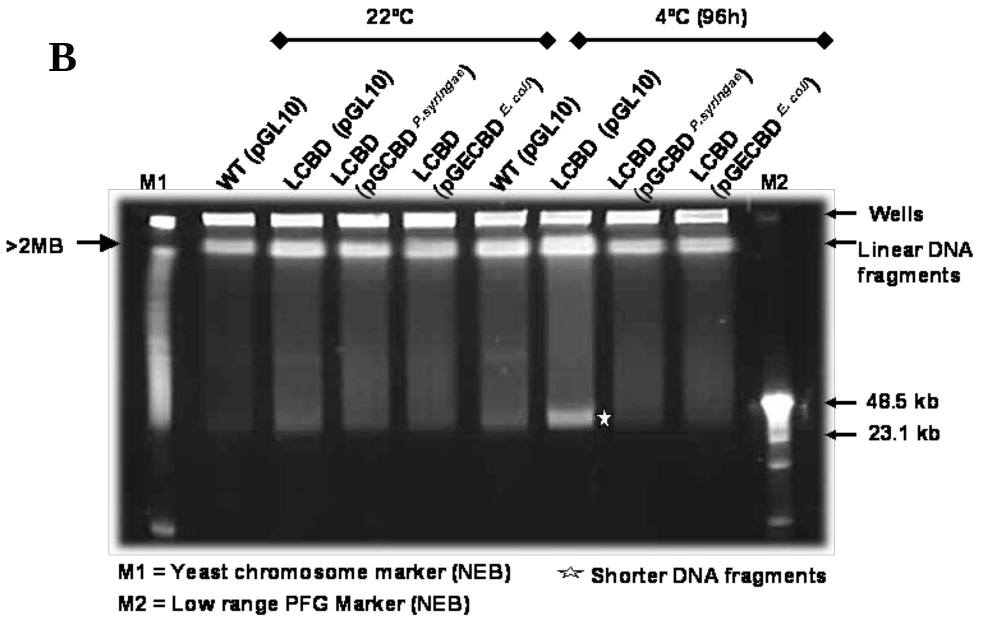

Supplementary* Figure *S2

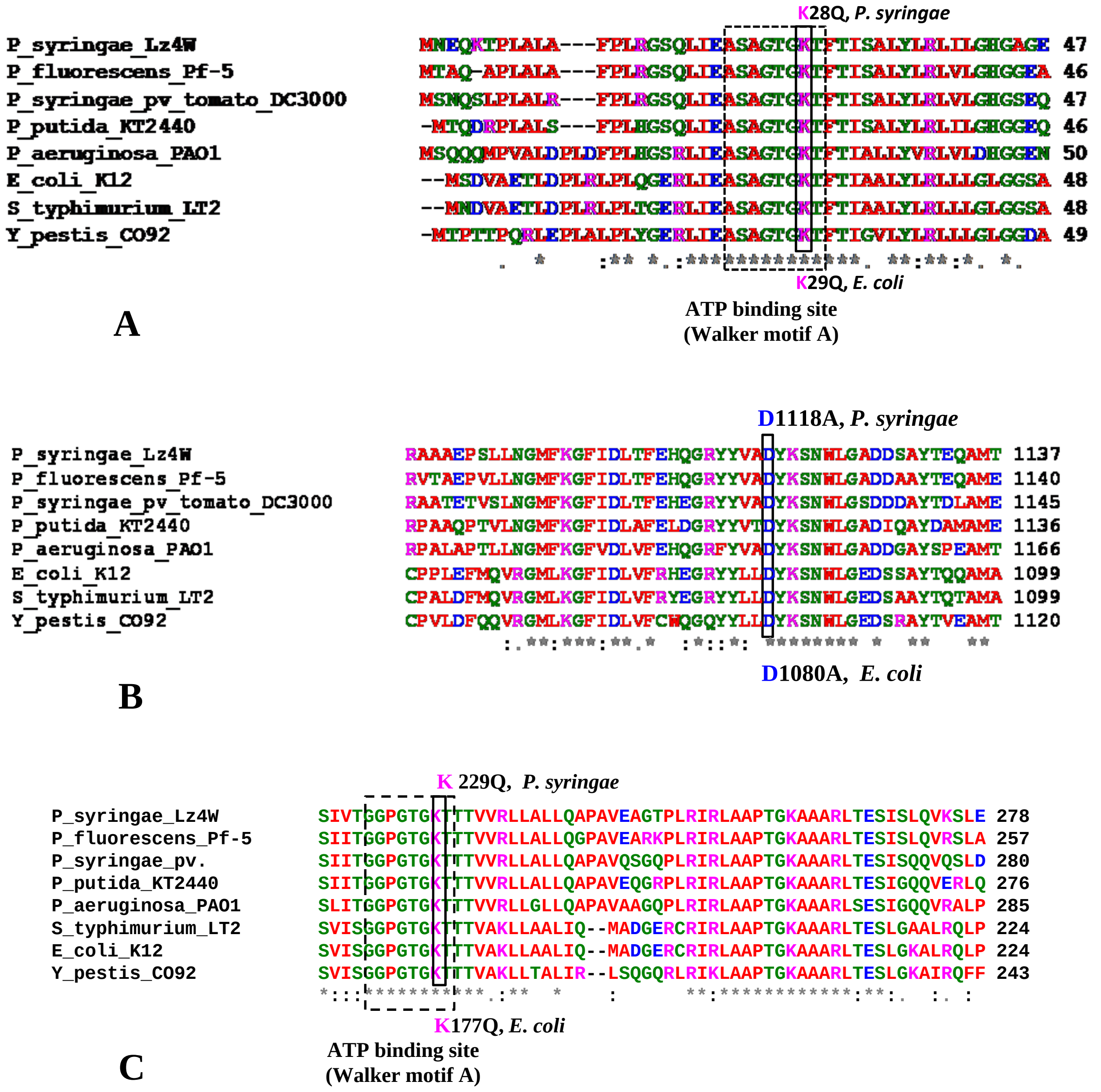

Supplementary Figure *S3*
